# Multiscale Modeling of Bio-Nano Interactions of Zero-Valent Silver Nanoparticles

**DOI:** 10.1021/acs.jpcb.1c09525

**Published:** 2022-02-08

**Authors:** Julia Subbotina, Vladimir Lobaskin

**Affiliations:** School of Physics, University College Dublin, Belfield, Dublin 4, Ireland

## Abstract

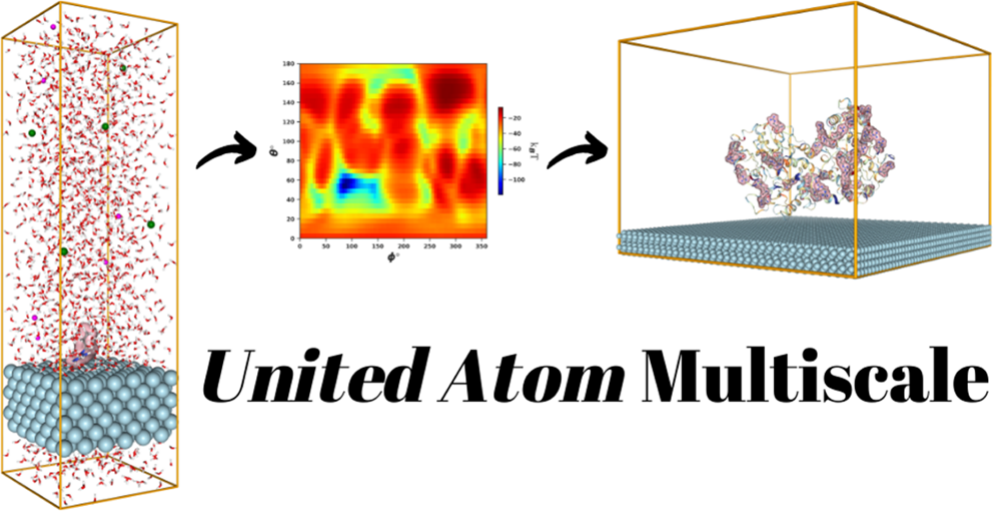

Understanding the specifics of interaction between the protein and nanomaterial is crucial for designing efficient, safe, and selective nanoplatforms, such as biosensor or nanocarrier systems. Routing experimental screening for the most suitable complementary pair of biomolecule and nanomaterial used in such nanoplatforms might be a resource-intensive task. While a range of computational tools are available for prescreening libraries of proteins for their interactions with small molecular ligands, choices for high-throughput screening of protein libraries for binding affinities to new and existing nanomaterials are very limited. In the current work, we present the results of the systematic computational study of interaction of various biomolecules with pristine zero-valent noble metal nanoparticles, namely, AgNPs, by using the *UnitedAtom* multiscale approach. A set of blood plasma and dietary proteins for which the interaction with AgNPs was described experimentally were examined computationally to evaluate the performance of the *UnitedAtom* method. A set of interfacial descriptors (log *P*^NM^, adsorption affinities, and adsorption affinity ranking), which can characterize the relative hydrophobicity/hydrophilicity/lipophilicity of the nanosized silver and its ability to form bio(eco)corona, was evaluated for future use in nano-QSAR/QSPR studies.

## Introduction

The antimicrobial properties of metallic silver are well known for centuries.^1^ During the last few decades, nanosilver (Ag particulate matter with at least one dimension less than 100 nm) has found numerous applications in several fields.^2,3^ In medicine,^4^ it was exploited as an antibacterial, antifungal, antiviral, and anti-inflammatory agent. The antimicrobial properties made nanosilver a popular antifouling coating for implants, catheters, and other surfaces contacting with biological fluids, as well as a frequent additive ingredient for textile and cosmetic products. Additionally, silver nanoparticles (AgNPs) attracted great interest for bio- and chemosensing applications^5−7^ as they possess distinct and tunable plasmonic characteristics (a localized surface plasmon resonance). However, such popularity of AgNPs caused their increased occurrence in the biosphere, and this situation has raised many concerns due to their potential toxicity.^8,9^

The potency of nanosized silver is linked to its advanced reactivity arising from the higher surface area-to-volume ratio when compared to the bulk material. After immersion of AgNPs into biological fluids (e.g., blood serum and plasma, mucus), they interact with the fluid content to form various agglomerates.^10^ Depending on the exposure pathways (e.g., at the contact area of blood with medical instruments, via air pathways, or skin), a multitude of biochemical and biophysical responses can be invoked.^11^ Layering blood plasma proteins on the surface of the nanomaterial^12^ is the most common response of the organism against the nanosized intruder. Masking the nanomaterial (NM) with such biocorona provides a pristine NM with “a new identity”^13^ which helps it to stay undetected below the cellular defence radars and allows it to penetrate the membrane into the cell, where it can interact with intracellular constituents, for example, organelles, lipid bilayer, proteins, DNA, and other biomolecules. These interactions at the bio-nano interface may affect the NM toxicity, which is governed by a variety of factors (size and shape of NPs, material, surface chemistry of the material, type of cells, etc.).

Although toxicity is an undesirable feature of the nanosized material, it can be harnessed to prevent the growth of cancer cells or pathogenic bacteria. Specifically, NPs that can pass safely through the blood–brain barrier become attractive candidates for drug delivery and analytical nanoplatforms aimed to treat and detect inaccessible targets for cancers and neurodegenerative diseases.^14^

The high target specificity of such nanoplatforms, which ensures their low risk profile, can be achieved through a “safe-by-design” approach. Decorating NPs with molecular entities, selectively coupled to the extracellular elements of the tumor cell membrane [e.g., circular dichroism (CD) markers^15,16^], is a common strategy that helps to deliver NPs directly to the area with a high localization of tumor cells. Coupling of the molecular entities to the NPs can be done either via chemical bonding or by physisorption. In some cases, it was reported that physical adsorption is a preferred coupling mode as it resulted in higher uptake of NPs.^17^

**Figure 1:**
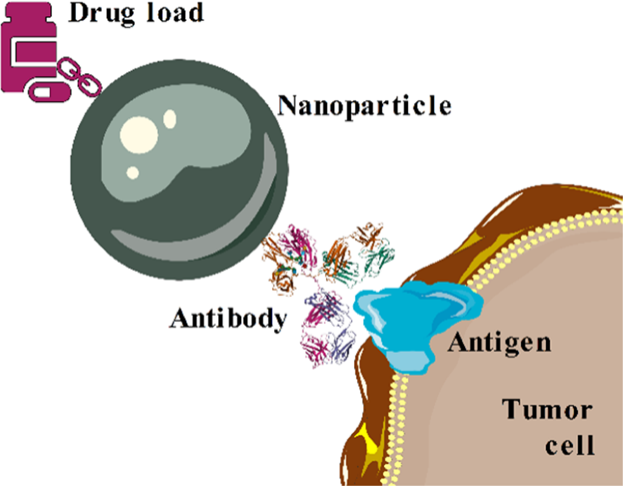
Working principle of the drug nanocarrier for cancer therapies. The figure was prepared by using Servier Medical Art^20^ service.

After the attachment, the NPs can damage the membrane of the tumor cell causing its death. Alternatively, cell death can be triggered by the drug which was preloaded to the NP (Figure 1). In this scenario, the NP acts as a vehicle delivering the antitumor reagent directly to the target (a nanocarrier^18,19^).

In both scenarios, finding a safe and optimal “drug load/coupled agent/NM” combination experimentally can be a daunting task as it requires systematic screening of the large libraries of proteins, ligands, and NMs. On the other hand, the prescreening for potential candidates can be performed *in silico*. Numerous computational approaches have been developed to evaluate binding affinities between various molecules with the help of rigorous computational protocols combining docking techniques and molecular dynamics (MD) simulations.^21−23^ However, despite their accuracy and veracity, brute-force techniques are hardly applicable for *in silico* prescreening of numerous protein adsorbates for NMs due to their significant computational cost. A typical computation of the protein adsorption energy using all-atom MD models entails introducing enhanced sampling techniques to address the vast number of degrees of freedom of the protein as it can undergo conformational changes in the globular structure upon adsorption onto a solid surface. Given the size of such a system and the high energy barriers associated with such an interaction,^24−27,37^ getting a representative sampling for protein adsorption onto solid surfaces becomes a profound task.

To bridge the length and time scale gap, several coarse-grained (CG) models to describe the bio-nano interaction have been introduced. In particular, we proposed a bottom-up multiscale *UnitedAtom* (*UA*) approach to estimate protein adsorption energies onto NPs,^28−30^ where the globular protein was represented by a collection of one-per-amino-acid CG beads. The total interaction potential between the protein and NP is evaluated by summation of all types of molecular interactions between the CG beads and the NPs. The total *UA* adsorption energy of the protein onto NM was divided into two major contributions arising from the short-range forces, computed with a high resolution using all-atom MD simulations, and the long-range forces, evaluated via CG potentials. The *UA* model has been already justified and applied to predict protein binding energies for titania and gold NPs.^31,32^

In the current work, we present an extension of *UA* model parametrization for predicting the adsorption affinities for zero-valent silver NPs.

The remainder of the paper is divided into three sections. In the first one, we will give a high-level overview of the *UA* model. In the second part, we will present results of the parametrization of *UA* short-range surface interaction potentials for three types of face-centered cubical (fcc) Ag structures. Thirty-two CG beads, representing amino acid (AA), carbohydrate, and lipid fragments, were parametrized. This set of parameters is sufficient to prescreen *in silico* adsorption affinities for various types of proteins (including glyco- and lipoproteins). Last, we will discuss the computed adsorption characteristics for several dietary and blood plasma proteins and compare these predictions with the available experimental data.

## Methods and Materials

### *UA* Model

In this section, we will revisit the theoretical backgrounds of the *UA* model which was formulated and justified in earlier publications.^28−30^ In the *UA* model, only physical adsorption of biomolecules on NM surfaces is considered. It is assumed that both parts representing the bio-nano interface exist in the aqueous medium containing counterions or salts with a constant concentration corresponding to standard physiological conditions. The aqueous medium (water) is represented implicitly. The protein is treated as a rigid body with CG beads that has no internal movements. In the simplest approximation of the *UA* model, the NP is also treated as the rigid flat surface, sphere, or cylinder with a defined thickness/radius.

The total interaction potential between the NP and the protein for a given configuration is written in a pairwise-additive way via individual nonbonded interaction potentials corresponding to each CG AA bead. These potentials depend on the distance *d*_*i*_ between centers of mass (COMs) of the NP and each AA. At the same time, the distance *d*_*i*_ depends on the orientation of the whole protein with respect to the NP surface, which is defined by two rotational angles θ and ϕ relative to the initial protein orientation defined in the PDB file
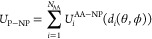
1

The CG interaction energy for each AA is the sum of nonbonded (van der Waals, dipolar, and excluded volume) and electrostatic terms
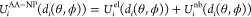
2

The electrostatic interaction potential between the NP and AA, which implicitly accounts for properties of the environment, for example, ionic strength *I*, buffer composition, salt concentrations *c*_*i*_, and the dielectric constant of solvent ε, is defined as follows:
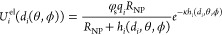
3where φ_s_ is the electrostatic surface potential of the NP, *R*_NP_ is the NP radius, *h*_*i*_(*d*_*i*_,θ,ϕ) is the distance between the COM for AA and the surface of the NP,  is the inverse Debye length,  is the Bjerrum length, and *I* for ions of valencies *z_i_* is given by
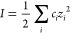
4

The pH factor is excluded from consideration in this work as we assume a neutral pH corresponding to standard physiological conditions in all calculations. However, preliminary checks on the protonation states of the protein at pH = 7.0 submitted into the *UA* calculations are recommended as CG beads corresponding to protonated/deprotonated AAs are included in the parameterization set.

Although the NP is assumed to be homogeneous in the *UA* model, nonbonded interactions of AAs with the NP’s inner core and surface parts are modeled at different resolutions. The nonbonded potential is split into two parts, representing the nonbonded interaction of each AA with the surface (*U*_*i*,s_^nb^) and the core (*U*_*i*,*c*_^nb^) of the NP. The nonbonded potential (*U*_*i*_^nb^) for each AA depends on the distance *h*_*i*_(*d*_*i*_,θ,ϕ) between the NP surface and the COM for AA
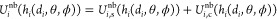
5

**Figure 2:**
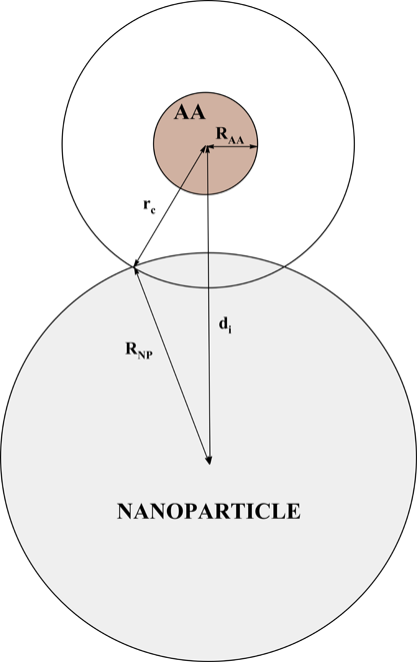
Definition of the cutoff distance *r*_c_ in the *UA* model (lens model^28^).

The boundary between the core and surface regions of the NP is defined by a cutoff distance *r*_*c*_ (Figure 2), and any nonbonded interactions below that distance (e.g., short-range surface potential *U*_*i*,s_^nb^(*h*_*i*_(*d*_*i*_,θ,ϕ))) are evaluated by all-atom MD simulations as a potential mean force (PMF). Typical value for *r*_c_ is in the range 1.0–1.2 nm, and its choice depends on the selected force field applied for the calculation of the short-range surface potential.

Nonbonded interactions outside the cutoff distance (long-range core potential *U*_*i*,c_^nb^), arising from dispersion forces acting through the water medium between the NP core and the *i*-th AA of radius *R*_AA_, are approximated by the Hamaker potential^33^
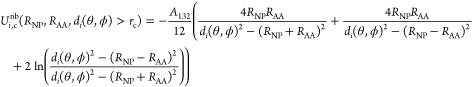
6where *A*_132_ is a Hamaker constant for materials 1 (NM) and 2 (*i*-th AA) interacting through solvent 3. It can be calculated^33^ from the experimentally measured dielectric permittivity ε_j_ and the refractive index *n*_*j*_ of participating phases *j* = 1, 2, 3:

(a) If materials 1 (AA) and 2 (NP) are both dielectric, then

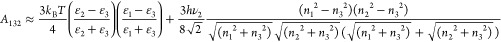
7

(b) For piezoelectric/conducting/semiconducting NM 2

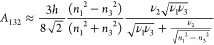
8where *v*_*i*_ is an electronic absorption frequency at maximum absorbance peak in the UV spectra of corresponding dielectric materials; for the conducting material, *v*_*i*_ is a plasma frequency (e.g., *v*_2_ in Eq. 8), and *h* is the Planck’s constant.

At distances shorter than *r*_c_, the short-range core potential *U*_*i*,*c*_^nb^(*h*_*i*_(*d*_*i*_,θ,ϕ)) should be corrected to avoid double counting of the nonbonded interactions encoded in the PMF computed along the surface separation distance (SSD), defined^34^ as *h*_*i*_ = *d*_*i*_ – *R*_NP_ by adding
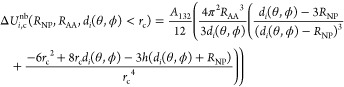
9

The final summation of all terms for individual AA described above over the protein sequence yields the interaction energy for a given (θ_*k*_,ϕ_*l*_) orientation of the protein positioned at the *z*-distance between the COM of the NP and the COM of the whole protein. Sampling over all possible protein orientations defined by rotational angles (θ_*k*_,ϕ_*l*_) and scanning along *z*-distance produces a set of *U*_P–NP_(*z*,θ_*k*_,ϕ_*l*_) potentials corresponding to a multitude of configurations for an NP–protein complex. The mean interaction energy for a particular orientation (θ_*k*_,ϕ_*l*_) within a corresponding distance interval 0 ≤ *z* ≤ *a*(θ_*k*_,ϕ_*l*_) can be evaluated as follows:

•for the protein interacting with a flat slab

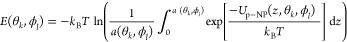
10

•for the protein interacting with a spherical NP

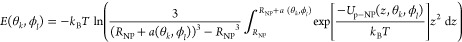
11

Averaging the mean interaction energies *E*(θ_*k*_,ϕ_*l*_) over all possible configurations (θ_*k*_,ϕ_*l*_) yields the final mean adsorption energy *E*_ads_^A^ (arithmetic mean of the values obtained at all the sampled protein orientations).^35^ Alternatively, the averaging can be performed via canonical averaging with Boltzmann weighting factors *P*_*kl*_ yielding the average adsorption energy *E*_ads_^B^
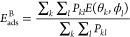
12
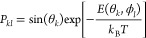
13

### Reconstruction of a Short-Range NP-AA Surface Potential ***U***_*i*,s_^**nb**^(***h***_***i***_(***d***_***i***_,**θ**,ϕ)) from the PMF

As was mentioned in the previous section, the short-range surface potentials for individual CG AA beads should be precalculated as PMFs at the all-atom resolution. Various techniques exist for an accurate evaluation of the binding free energy for molecules.^36−39^ Recently, an adaptive well-tempered metadynamics (AWT-MetaD)^40^ has become popular to obtain interaction free energy as PMFs. Its accuracy is based on enhanced sampling techniques of configurational space which can be provided at a reasonable computational cost. The application of this method to study interfacial systems was previously described in ref (34) for the adsorption of biomolecules on the TiO_2_ (100) surface. The reported protocol was closely followed in the current work with some adjustments related to the use of a different force field. All MD simulations in the present work were performed in GROMACS.^41^

Three different fcc configurations, namely, (100), (110), and (111), were constructed by CHARMM-GUI/Nanomaterial Modeler^42^ tool. The slab thickness for each configuration varied from 1.012 to 1.180 nm. The simulation boxes for AWT-MetaD runs were constructed in the following way: the biomolecule fragment was placed above the silver slab 1.5 nm away from the surface, and the system was solvated by TIP3P water and neutralized by 0.15 KCl. The final dimensions of the starting simulation boxes were approximately 2.4 nm × 2.4 nm × 8.5 nm.

The simulation systems were subject to two subsequent preliminary unbiased equilibrations for 30 ns each under *NPT* and *NVT* ensemble conditions to obtain proper densities and dimensions. The temperature was kept constant at 300 K. The pressure was set at 1 bar. The relaxation time constant for the Nose–Hoover thermostat for the *NVT* ensemble was 5 ps, while Berendsen’s weak coupling thermostat and barostat were invoked for *NPT* simulations. Periodic boundary conditions were applied for all MD simulations in this work.

Pre-equilibrated systems then underwent AWT-MetaD simulations under *NVT* conditions for at least 600 ns to obtain an adequate sampling, yielding accurate PMF profiles. The AWT-MetaD simulations were performed by GROMACS^41^ coupled with PLUMED^43^ software. The collective variable (CV) *h*_*i*_ for one-dimensional adsorption PMFs, or the SSD, was defined as in work^34^ and was sampled in the range between 0.0 and 2.0 nm. The temperature for biased simulations was set at 300 K. Gaussian hills were added every 0.5 ps starting with an initial height of 2.5 kJ/mol. The bias factor was *f* = 20.

Particle Mesh Ewald scheme was used for long-range electrostatics treatment in all simulations. The cutoff distance (1.2 nm), recommended^41^ for CHARMM force field parameters, was used for treating short-range van der Waals interactions and long-range electrostatics.

The convergence of AWT-MetaD runs was controlled via the evolution of three parameters during the simulation: (1) the CV SSD, (2) the heights of the hills, and (3) the free energy difference between the minimum located on PMFs and the global minimum (the lowest state). The Metadynminer R package^44^ was utilized for this purpose.

### NM Hydrophobicity Descriptor as a Function of the Heat of Immersion

To understand the driving forces behind biomolecular adsorption onto inorganic NMs, it is instructive to quantify the adsorbent’s interaction with the solvent. The enthalpy of wetting (often presented as the heat of immersion) is the enthalpy change associated with immersing a solid in a wetting liquid, and it can be considered as a measure of hydrophobicity/hydrophilicity of the NM. The enthalpy of immersion can be measured experimentally by calorimetry or predicted computationally. A convenient computational method presented in ref (45) was based on enthalpy difference estimates for three systems: solid slab immersed in the liquid, the same solid slab in a vacuum, and a box of the same number of molecules of liquid as in the slab-liquid system. Then, the immersion enthalpy can be calculated as
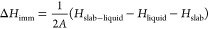
14where *A* is the area of the interface in the slab–liquid system.

When characterizing an NM for biomedical applications (e.g., implant or dental filling biocompatibility) and estimating its toxicity, it is essential to understand the relative affinity of the NM to the lipid bilayer (lipophilicity) and physiological aqueous liquids (hydrophilicity). In vHTS/QSAR studies on druggability of small molecules, the comparative hydrophilicity/lipophilicity of the drug candidate is usually described in terms of octanol–water partitioning coefficient log *P*,^46,47^ which is a logarithm of the ratio between solute (drug) concentrations in two phases of a biphasic system “*n*-octanol/water,” and can be evaluated computationally via an alchemical thermodynamic cycle based on relevant solvation free energies^48,49^
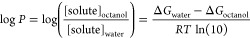
15

Inspired by this idea, we propose to quantify the relative hydrophilicity/lipophilicity of a solid crystalline NM as a function of relative enthalpy of immersion of a well-defined periodic NP slab in water and octanol, representing aqueous and lipid physiological phases
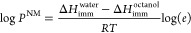
16

Such interfacial descriptors can be useful for predicting cell adhesion properties of materials, which in turn is known to correlate with the biocompatibility of the NM. A negative value of log *P*^NM^ then will be indicative of the material with a higher affinity for the aqueous phases (hydrophilic), while a positive value of the descriptor will point to the material with an affinity to hydrophobic (e.g., protein corona) and lipophilic (e.g., cell membrane) environments. It should be noted that definition of log *P*^NM^ descriptor given in eq 16 does not include entropic terms corresponding to any structural reorganization in slab/liquid systems which actually may occur upon mutual interaction. However, for comparative nanoinformatics studies of materials with similar solvation behavior, this descriptor can be useful for obtaining relative hydrophilicity/lipophilicity rankings.

To calculate the Δ*H*_imm_ values in water and octanol for all fcc configurations, the corresponding systems (NP slab, octanol and water boxes, NP slab in water and octanol) were pre-equilibrated for 30 ns and simulated further for 350 ns under *NPT* conditions to ensure proper statistics for collected energies. All other parameters were as described in the previous section. The energy files corresponding to collected trajectories were analyzed to obtain enthalpy averages and error estimates using block averaging (*gmx energy* and *gmx analyze* modules in GROMACS).

### Selection of Force Field Parameters for Interfacial MD Simulations

Various force fields, polarizable and nonpolarizable, were proposed by different authors^50−55^ to model interfacial processes occurring at the surface of the noble metal. In the current work, the nonpolarizable INTERFACE force field by Heinz et al.^53,56^ was used for the metallic “nano” part of the system, while CHARMM36 force field parameters^57^ were applied to the remaining “bio” part.

Inclusion of polarization effects implicitly via rigorous parametrization in additive INTERFACE/CHARMM36 FF may result in some inconsistencies^58^ when FFs are applied to model the bio-nano interface phenomena (e.g., image charge interactions). However, not many existing polarizable force fields cover a large number of inorganic materials and biomolecules at the same time and are suitable for ongoing systematic consistent parametrization of NMs for the *UA* model. In principle, accurate treatment of the image charge interactions at the metal interface can be achieved by *a posteriori* inclusion of the polarization effects,^59^ although at a significant computational cost. In the current study, the exclusion of polarizable effects for calculation of short-range potentials for adsorption of single AA did not substantially impact the outcome—the relative adsorption affinity rankings of proteins (protein abundancies) calculated by the *UA* approach, especially for the proteins with a net charge. However, the use of polarizable FFs might be more beneficial and additional calculations might be needed to evaluate the error arising from the choice of the force field for the noble metal NMs.

**Figure 3:**
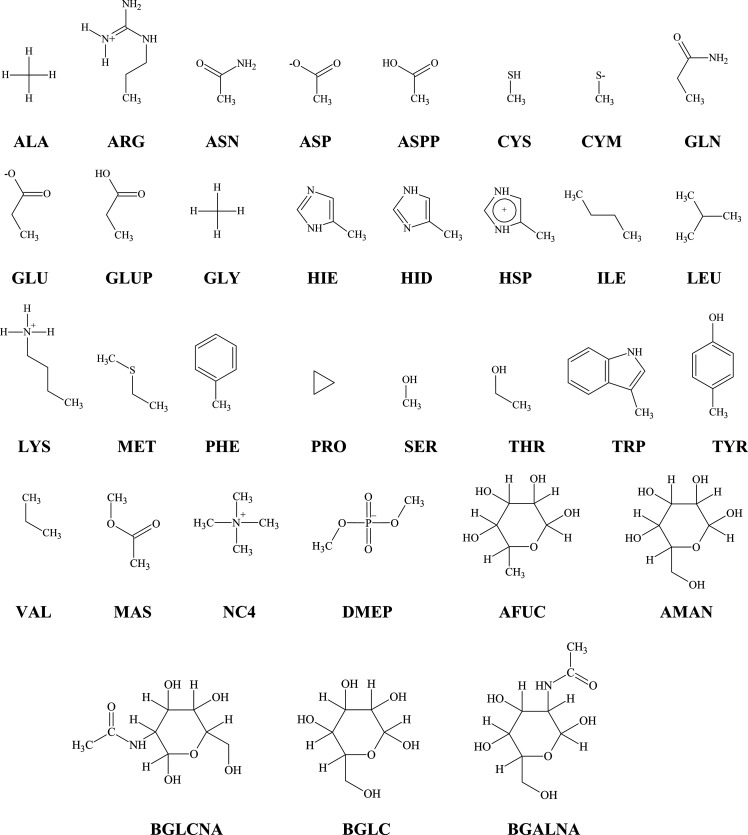
Side chain analogues of AAs, carbohydrates, and lipid fragments for building CG models of proteins.

The rationale for selecting the CHARMM36 FF for the “bio” part was based on two factors: (a) the wide range of residues represented in the force field collection and (b) the scheme used for grouping atomic charges within the single residues. It should be noted that the *UA* scheme has an additional assumption for the protein representation: the term corresponding to the interaction of the protein backbone with the NP is excluded from the short-range AA–NP potentials; only terms corresponding to the interaction of side chains of AAs (SCAs) with the NP are considered. To simulate only the side-chain fragments, the CHARMM36 FF topology files for AAs were adjusted: atoms in the backbone group were replaced by a neutrally charged hydrogen atom. The short-range *UA* potential for GLY AA was not calculated; it was replaced by NM–ALA potential instead. For convenience, a CHARMM FF residue naming convention was kept to name CG beads in multiscale calculations (Figure 3).

### Three-Dimensional Structures for Proteins

The structures of proteins studied in this work were obtained from Protein Data Bank (PDB) and prepared for further modeling by CHARMM-GUI/PDB Reader.^60^ Original protonation state corresponding to pH = 7 was employed. No additional structural refinement by MD was done. Previously we have shown^31^ that positional root-mean-square deviation (rmsd) fluctuations for C_α_ atoms of the AA residue up to 0.1 nm did not significantly alter the mean binding energy for the whole protein. However, the real magnitude of rmsd fluctuations associated with adsorption may be different for different families/types of proteins. Proteins selected for the current study were characterized with moderate structural changes associated with binding to the AgNPs and kept their biological functions upon adsorption (see cases 1–8 in the Supporting Information). In this scenario, a rigid protein structure with coordinates preserved from the PDB file should be sufficient to obtain a descriptive picture of the mean adsorption energy. For the proteins, where unfolding upon binding to the NP can be significant, more accurate estimates for the mean adsorption energies can be achieved by using a set of perturbed rigid protein structures. The improvement of the *UA* model with respect to protein flexibility is planned for the future development.

## Results

### Short-Range Surface Adsorption Potentials of Carbohydrates, Lipid Fragments, and AA Side Chains

**Figure 4:**
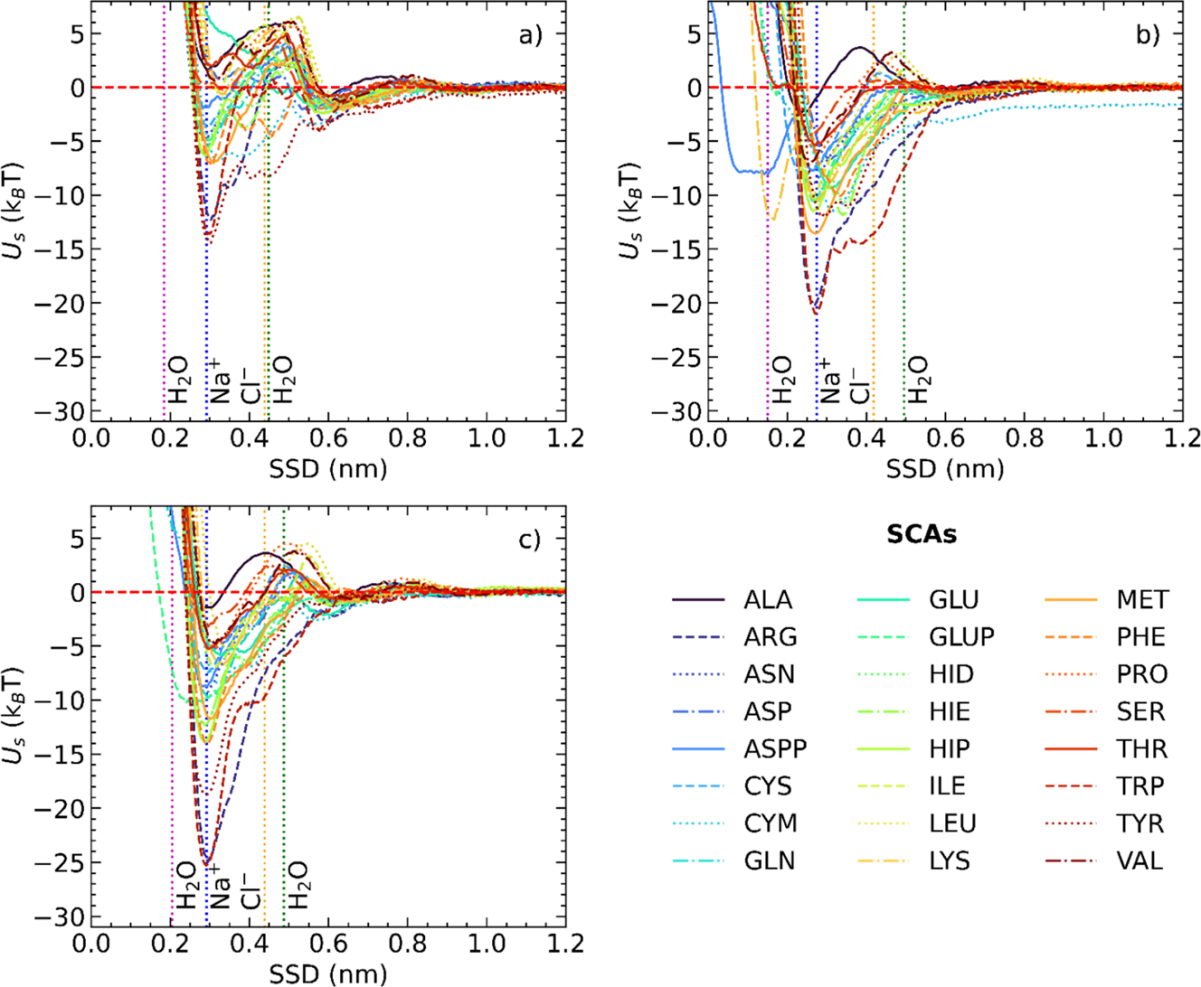
Short-range surface adsorption potentials *U*_*i*,s_^nb^(*h*_*i*_(*d*_*i*_,θ,ϕ)) of AA side chain analogues onto AgNPs obtained with AWT-MetaD simulations. (a) fcc (100) surface. SCAs can stay in the bulk water region as well as can pass through the second adlayer of water. As a result, the weakest adsorption of SCAs was predicted for this fcc configuration. (b) fcc (110) surface. SCAs occupy a region closer to the NP surface and between two adlayers of H_2_O. This leads to stronger adsorption of SCAs; however, adsorbates are unable to replace water molecules from the first water shell around the NP. (c) fcc (111) surface. Vertical dashed lines correspond to the positions of maxima in the density profiles for water molecules and counter around the metallic slab.

**Figure 5:**
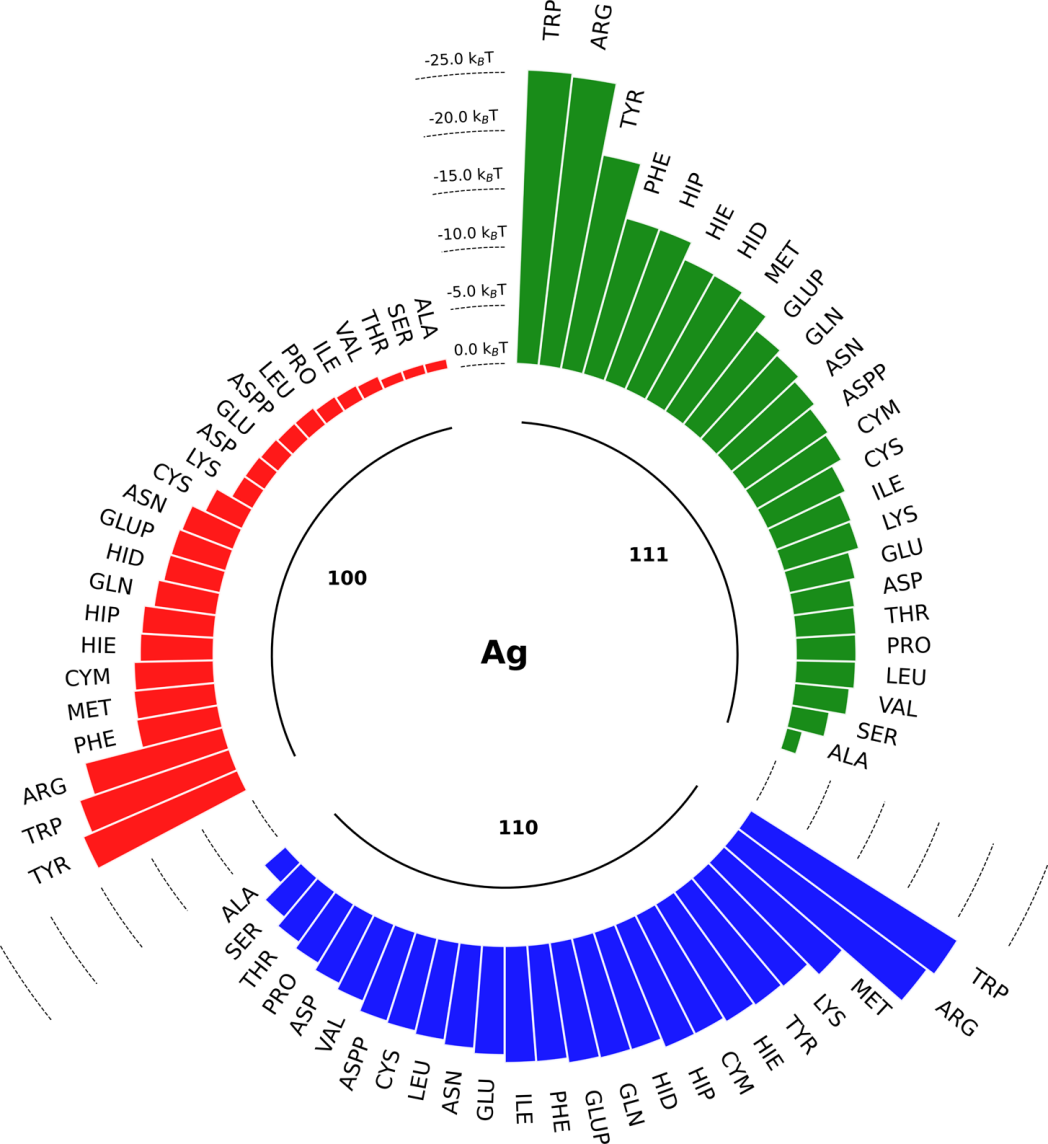
Adsorption energies *E*_min_ for biomolecules (in *k*_B_*T*). Aromatic and positively charged residues have a slight preference for adsorption onto silver surfaces. The distinct preference trend for aromatic SCAs to be adsorbed onto the Ag(111) surface was noted. On average, SCAs have a preference to bind to the Ag(110) facet, followed by Ag(111) and Ag(100). The weakest binding was predicted for the Ag(100) facet as a result of the lower hydrophobicity of this fcc configuration.

In total, 96 PMF profiles for adsorption of biomolecules were calculated to obtain SCA’s short-range surface potentials, *U*_*i*,s_^nb^(*h*_*i*_(*d*_*i*_,θ,ϕ)), for three fcc configurations (see Figure 4 and the dataset^61^). The majority of PMFs converged within 400 ns of production run (see Arg-Ag(110) system example at Figure S2 in the Supporting Information). Larger molecules required slightly longer simulation (up to 600 ns) to obtain reasonable configurational sampling. Values of adsorption energy Δ*F*_ads_, calculated by numerical integration of PMF curves, are collected in Table S1 in the Supporting Information. In general, biomolecules prefer to be adsorbed onto (111) or (110) facets of AgNPs: the calculated mean values of adsorption energy Δ*F*_ads_ per SCA were −1.94 *k*_B_*T*, −5.83*k*_B_*T*, and −6.03*k*_B_*T* for Ag(100), Ag(110), and Ag(111) slabs, respectively. The same outcome was obtained by comparing the energies of the lowest minima on PMF curves *E*_min_ (Figure 5). Aromatic residues were predicted to bind stronger to all configurations of Ag fcc. This trend was particularly evident for the Ag(111) surface. The preference for aromatic molecules to be bound to (111) noble metal surfaces was previously explained^56^ by the match between atoms of the hexagonal ring and epitaxial sites on the metallic plane. A strong interaction between Au(111) epitaxial sites and polarizable atoms (O, N, C) resulted in soft epitaxial adsorption.

The general binding prevalence of aromatic AAs was also established experimentally for the interaction of various di- and tripeptides with colloidal silver,^62^ where Phe, Tyr, and Trp sites demonstrated a stronger adsorption affinity. The authors also noted a high affinity of the amino group for the silver surface which resulted in peptides’ adsorption via the N-terminus. Such adsorption behavior was explained by the attraction between two polarizable dipoles, associated with an electron density of AgNP outer layer on one side and a delocalized π-system on aromatic rings or a charged terminus on the other side.^63,64^

A binding preference for linear molecules on (110) surfaces was previously reported^56^ and explained by better geometric alignment of polarizable atoms with epitaxial sites on an inorganic surface. Calculated binding energies for SCA support this observation (Figure 5): Lys was predicted to bind stronger to the Ag(110) surface than Phe. Aliphatic residues Arg and Met were also predicted to be a strong binder for all three facets (Figure 5).

At the same time, heteroatom-rich biomolecules, such as carbohydrates and dimethyl phosphate, have shown stronger binding affinities to AgNPs as compared to SCAs. For example, the mean adsorption free energy Δ*F*_ads_ for *N*-acetyl hexosamine BGALNA was calculated at −19.46*k*_B_*T*, −25.66*k*_B_*T*, and −27.40*k*_B_*T* for Ag(100), Ag(110), and Ag(111) crystal planes (Table S1, Supporting Information).

Predicted strong binding of carbohydrates to the silver surface can be related to antimicrobial potency of colloidal silver^65^ as sugars are important in maintaining the integrity of viruses and bacteria by supporting their membrane functions. In viruses, glycans are located on their outer surface and responsible for the attachment of the virus to the cell membrane of the infected host.^66^ Thus, blocking viral glycans by AgNPs should reduce the probability of host–virus association. In bacteria, the interaction of colloidal silver with peptidoglycans, composing bacterial cell walls, should also alter bacterial functions^67^ as glycans play a crucial role in the cellular pathways present in microbes.

The importance of the glycosylation state of proteins was also noted for the future nanocarriers rational design. The authors^68^ have shown that deglycosylation of the proteins in the corona of polymeric NPs significantly changed their cellular uptake. The deglycosylation of protein corona of silica NPs^69^ decreased the colloidal stability of NP–protein complexes and led to improved cell adhesion of the NP.

### Structured Hydration Shell around Silver NPs and Hydrophobicity of the NM

The shape of computed PMFs for SCAs was impacted by the character of interfacial processes occurring between the NP and the solvent. The water density profiles for the interfacial water layer obtained from MD simulations for the “slab–water” system suggested the existence of two regions with elevated water density found 0.15–0.20 and 0.45–0.49 nm away from the silver surface (Figure S3 in the Supporting Information). Analysis of MD trajectories showed that water molecules in the first adlayer have the direction of their dipole moments aligned with the positive direction of the normal to the metal surface as the cosine of the angle between these two vectors remains positive (Figures S4 in the Supporting Information) at approx. 0.25 and 0.6 nm (so the angle is −90° ≤β ≤ 90°). Such an orientation of water dipoles is in line with existing experimental data on a solvent organization for various colloidal NPs.^70,71^ The existence of adsorbed water layers on conductive surfaces is due to the image–charge interactions of water molecules with the metals.^72^

Examination of the ion density profiles also revealed the existence of two adjacent layers for sodium and chloride ions at 0.27–0.36 and 0.42–0.50 nm away from the metal surface. However, due to a relatively low volume concentration of ions, the peaks were less pronounced. The sodium ions are located slightly behind the first water adlayer at 0.15–20 nm, while the location of chloride ions almost overlaps with the second water adlayer at 0.45–0.49 nm (blue and yellow vertical dashed lines in Figure 4). This alignment indicates that sodium ions are taking positions next to the water oxygen atoms of the first adlayer and chloride anions are incorporated in the network of water molecules of the second adlayer. A similar arrangement was predicted for interfacial aqueous LiCl electrolyte solution at the platinum electrode plane (fcc 100) under zero applied potential.^72^ The estimated energy cost of desorption of the first water adlayer from the Pt surface was ∼10*k*_B_*T*, and the cost of desorption of Li^+^ from the water layer was about ∼3*k*_B_*T*. At the same time, no barrier was predicted for the exchange of molecules between the bulk water and the second hydration shell around the Pt electrode.

The existence of charged ionic adlayers next to the NP surface suggests that adsorption patterns for biomolecules can be significantly impacted, especially when higher ion concentrations and polyvalent ions are present in the media as a result of interfacial polarization alterations predicted for polarizable NPs (e.g., noble metals).^73^

Computed PMF profiles (shown in Figures 4 and S1 in the Supporting Information) had no adsorption minima next to the NP surface (SSD < 0.15 nm), suggesting that adsorbates were not able to expel water molecules from the first hydration shell around the AgNP. However, they were able to penetrate through the second interfacial water layer. The adsorption of protonated Asp residue onto the Ag(110) surface was the only exception showing the existence of two equal-depth minima preceding and following the position of the first hydration shell.

In the case of the Ag(100) surface, an additional adsorption minimum for some of SCA appeared at ca. 0.5–0.6 nm, suggesting that biomolecules may also remain in the solvent bulk without passing through the second hydration layer (Figure 4). This behavior resulted in the weaker binding of biomolecules to the Ag(100) surface in general (Figure 5) and can be associated with higher hydrophilicity of the Ag(100) surface as compared to Ag(110) and Ag(111) surfaces.

Various quantities have been used in the literature to characterize engineered NPs by their hydrophilicity/hydrophobicity,^74−76^ for example, contact angles, surface free energies, heat of immersion,^77^ or octanol–water affinity coefficients (KA_OW_). Nonetheless, their applicability for the characterization of a large dataset of engineered materials can be a daunting task not only due to time-consuming analytical techniques but also due to the inconsistency of measured results (e.g., surface energies for AgNPs^78^). Computational tools predicting those characteristics are available but also susceptible to the same issues. For example, inconsistent estimates for the surface free energies for silver slabs in *vacuum* obtained at a different level of density functional theory approximation have been published.^79−81^

We used MD simulations with eq 14 to evaluate the immersion enthalpies for three silver surfaces as described above. The calculated water immersion enthalpies for silver slabs (Table 1) indicated a slightly more hydrophilic character of the Ag(100) facet as compared to Ag(110) or Ag(111). Thus, hydrophobic molecules (e.g., proteins) will be less likely to replace water molecules from the water shells of the Ag(100) surface, and they will rather be adsorbed on (110) or (111) facets. At the same time, the immersion enthalpies calculated for “slab-octanol” systems point out a stronger interaction of AgNPs with hydrophobic/lipophilic matter, rather than with aqueous environments (log *P*^NM^ > 0). It means that AgNPs will tend to form biocorona with lipids and bind to the cell membrane.

**Table 1:** Surface Descriptors of Hydrophobicity/Hydrophilicity for fcc Ag

surface descriptors	Ag(100)	Ag(110)	Ag(111)
immersion enthalpy^a^ in pure water (kJ/mol nm^2^)	–276.70 ± 1.0	–206.7 ± 0.5	–204.6 ± 0.5
immersion enthalpy^a^ in octanol (kJ/mol nm^2^)	–287.0 ± 3.0	–289.0 ± 5.0	–308.0 ± 5.0
log *P*^NM^^b^	6.04	14.49	18.13

a Immersion enthalpies computed in the current work. The lower the enthalpy, the higher the preference for the material to be wetted with the selected solvent. The immersion enthalpy in water provides the measure of hydrophilicity. The immersion enthalpy in octanol provides the measure of lipophilicity.

b Relative measure of hydrophobicity/lipophilicity calculated as in eq 16.

### Multiscale CG Modeling of Protein Adsorption on a Silver NP

The thermodynamics and kinetics of protein adsorption process onto pristine AgNPs have been previously addressed for various blood plasma, milk, and other dietary proteins:^82−93^ for example, bovine (BSA, PDBID: 3V03) and human (HSA, PDBID:1AO6) serum albumins, bovine (BHb, PDBID: 1FSX) and human (HHb, PDBID: 1GZX) hemoglobin, papain (PDBID: 9PAP), bromelain (PDBID:1W0Q), lysozyme (PDBID: 1AKI), and bovine lactoferrin (BLf, PDBID:1BLF). Reported experimental metrics from isothermal titration calorimetry (ITC) experiments for listed proteins was used to assess the predictive power of the *UA* method. The adsorption of all proteins was reported to be exothermic, associated with negative free Gibbs energy values (Table 2). According to the CD spectra, selected proteins experience relatively small changes in the globular structure upon interaction with AgNPs: observed loss of α-helical content was ∼3–10%.^86,87,90−94^ Based on that, the “rigid body” approximation applied in the *UA* scheme should not cause a significant error in predicting adsorption affinities, arising from inaccurate information on protein coordinates.

**Table 2:** Experimentally Measured Free Energy of Adsorption Δ*G*_ads_ vs Calculated *E*_ads_ for Selected Proteins

					***E***ads, kJ/mol	
PDB ID	total charge, *e*	R(NP), nm	**ζ**-potential, mV	Δ***G***_**ads**_, kJ/mol	***E***_**ads**_^**A**^	***E***_**ads**_^**B**^
1BLF(93)	13	18.0	–28.1	–81.59	–34.47	–275.63
1W0Q(88)	5	40.0	–6.0^95^	–72.85	–27.61	–204.78
9PAP(88)	9	40.0	–6.0^95^	–59.76	–37.40	–213.71
3V03(94)	–32	40.0	–6.0^95^	–39.49	–30.89	–175.01
1AKI(89)	8	40.0	–6.0^95^	–28.71	–32.33	–169.79
1AO6(90)	–30	43.0	–6.0^95^	–22.14	–27.87	–154.70
1FSX(87)	2	10.0	–12.5^96^	–19.10	–23.54	–111.46
1GZX(86)	2	15.0	–15.5	–14.43	–24.54	–112.36

The experimental parameters for NP sizes and ζ-potentials were invoked in the *UA* scheme to calculate the average adsorption energies (*E*_ads_^A^ and *E*_ads_^B^) and to predict the lowest energy configurations of adsorption complexes. The inconsistency in reporting experimental parameters for protein adsorption experiments should be specially mentioned.^97^ For example, no information was available for ζ-potential of 40–43 nm-sized AgNPs used in studies.^88−90,94^ For these instances, the ζ-potential reported^95^ for the 40–70 nm distribution of spherical AgNPs synthesized in the presence of poly(*N*-vinylpyrrolidone) (PVP) was used (−6 mV). Although the polymeric capping should alter the ζ-potential of pristine NPs, the authors of the study suggested that impact of the PVP layer on particles’ properties should be minimal. Similarly, no ζ-potential was reported in the study of BHb adsorption.^87^ For this case, the measured value^96^ for 4–12 nm distribution of biosynthesized AgNPs was applied (−12.5 mV). Furthermore, the application of standard Langmuir binding models for extracting thermodynamic parameters from ITC experiments for protein binding results in the energy values that cannot be compared directly to the computed free energies.^98^ We should note that adsorption energies *E*_ads_ calculated by the *UA* approach are not free energies by construction. We will discuss this further in the Discussion section.

AgNPs used in experiments exhibit various types of crystal facets. Assuming that all facets equally contribute to protein adsorption, the final values for predicted absorption energies *E*_ads_^A^ and *E*_ads_^B^ (see Table S2 in the Supporting Information) were calculated as an arithmetic mean over all three facets (100), (110), and (111).

The resulting heatmaps for protein adsorption onto three facets of AgNPs are shown in Figure S5 in the Supporting Information. The calculated adsorption footprint of the same protein on different fcc facets remained similar, with only minor differences corresponding to the appearance/disappearance of alternative orientations of the protein. As it was mentioned earlier, the calculated adsorption energies for individual SCAs at short ranges were almost equal for (110) and (111) facets (−5.83*k*_B_*T* vs −6.03*k*_B_*T* on average per residue, Table S1 in the Supporting Information); however, a distinct preference for protein binding to the Ag(110) surface emerged after inclusion of long-ranged energy terms and as a result of cooperative effects (Table S2).

Proteins, containing glycosylated AAs in their structure, for example, bromelain and bovine lactoferrin, were predicted to be the strongest binders to AgNPs (Table 2), yet the contact between the NP surface and these proteins in the lowest energy adsorption complexes occurred through the charged residues and not via glycan fragments (Figure S6 and cases 6 and 8 in the Supporting Information). Positively and negatively charged residues (Arg, Lys, Glu, and Asp) were the most common contacts in the proximity of the metallic surface (Figure S6 in the Supporting Information). On the contrary, aromatic residues predicted to be the strongest individual binders, for example, Trp and Thr, did not make direct contact with Ag atoms in protein–NP complexes. This prediction did not contradict the experimental data from synchronous fluorescent spectrometry (SFS) for protein adsorption which suggested the change of microenvironment for aromatic residues upon protein adsorption in the case of BSA, HSA, bromelain, papain, lysozyme, and lactoferrin. Slightly different behavior in the SFS experiments was reported for hemoglobins, where tryptophane residues were acting as a binding site (see Supporting Information, cases 3 and 4).

However, it should be noted that in SFS experiments, proteins interact with the metallic surface in a partially unfolded state, and this cannot be captured by the *UA* model holding a “rigid body” approach. Hence, future modifications corresponding to the improved representation of the protein are required to increase the accuracy of the *UA* scheme. A detailed report on the calculated structures for NP–protein adsorption complexes can be found in Supporting Information, cases 1–8.

## Discussion

Adsorption of proteins on metallic surfaces is a complex phenomenon that proceeds through several common stages:^99^ (1) protein diffusion from the bulk of solvent to the interface, (2) protein anchoring to the second surface-bound water layer, (3) protein conformational rearrangement^100^ to achieve a better position and orientation within the second layer until (4) a lockdown state is reached, and final (5) protein self-diffusion at the water/solid interface to complete the adsorption. Modeling the details of such processes at the atomistic level for large proteins is currently unfeasible, so one needs to use multiscale approaches involving coarse-graining of the bio-nano interface. In addition, a data-driven approach (e.g., nanoQSAR and machine learning) for predicting such an interaction, where large sets of data are available, seems to be another favorable direction. To describe properly the interaction of biomolecules with inorganic materials, nanoQSAR models should include descriptors for proteins, NMs, and descriptors illustrating processes occurring at the solid/liquid interface (interfacial descriptors). Protein adsorption energies and quantitative measures of hydrophobicity/hydrophilicity, for example, enthalpies of wetting, can serve as interfacial descriptors for nanoQSAR models and in principle can be estimated computationally through MD and multiscale simulations. However, some considerations should be taken into account.

For example, as existing force fields usually do not provide a full set of parameters for many inorganic materials,^58^ the calculated values of wetting enthalpies for different materials obtained from different force fields cannot be compared directly. As a result, the hydrophilicity/hydrophobicity ranking of NMs based on these values might be inconsistent. The relative measure of hydrophilicity/hydrophobicity calculated within the same force field, such as log *P*^NM^, can perform better as an interfacial descriptor for the nanoQSAR models, providing the comparison between a range of NMs (e.g., prediction of cell adhesion responses for different NPs).

Computed adsorption energies *E*_ads_, either via the canonical averaging or simple averaging, showed adsorption ranking similar to the one predicted experimentally and can be used as efficient interfacial descriptors (Table 2). However, the exact mapping between Δ*G*_ads_ and the energy of adsorption *E*_ads_ is not possible for several reasons. First of all, the rigid body approximation of protein^28−30^ implemented in the *UA* model does not include energy terms related to structural rearrangements occurring at the bio-nano interface during the adsorption process (e.g., protein unfolding). It is anticipated that for proteins exhibiting insignificant changes in the globular structure upon adsorption these increments will be small. The structure of interfacial water also undergoes changes associated with cavity formation and protein insertion^101^ which takes place during the experiment. As it was mentioned above, the penalty for desorption of the first water adlayer from the noble metal surface is ∼10*k*_B_*T*, and this cost is comparable with experimentally measured adsorption free energies Δ*G*_ads_. Solvent reorganization effects related to the changes associated with a whole protein structure cannot be directly captured by the *UA* approach as a result of rigid body approximation and the implicit description of the solvent. However, these effects are partially included for individual side chains in the short-range surface potentials *U*_*i*,s_^nb^(*h*_*i*_(*d*_*i*_,θ,ϕ)) obtained via explicit all-atom MD simulations and should partially recover costs associated with solvent reorganization.

The implicit modeling of the environmental factors (e.g., variation in the ionic strength and pH) for the bio-nano interface may also lead to inaccurate estimates for electrostatic interactions between proteins and charged surfaces^102^ occurring at experimental conditions. Additional errors can also be inflicted by the neglect of image charge interactions between the charged residues and metallic surfaces in the current formulation of the *UA* model. The missing polarization energy contribution can be recovered either by using polarizable force fields at the atomistic MD level during the calculation of the PMFs^59^ or later at the CG level modeling by invoking a mean-field Poisson-Boltzmann theory.^103^ Based on the obtained results, the overall effect of not including these increments was relatively small as there were no large discrepancies between predicted and observed adsorption affinity rankings for highly negatively charged proteins (e.g., 3V03 and 1AO6, see Table 2).

It is also important to note that the *UA* method may overestimate the protein adsorption energies as it includes the contributions from all optimized arrangements of individual SCAs at the surface. However, in a real protein, where the translations and rotations of the side chains are constrained, not all arrangements of SCAs at the metal surface are reachable. This can explain the relatively high values of adsorption energies obtained by the ensemble averaging scheme (*E*_ads_^B^) where all configurations of NP–protein adsorption complex are included (eq 12). Due to this approximation in the sampling, the *UA* evaluated energy differs from the real adsorption free energy.

When applying the *UA* method to coated NMs, where coatings act as dispersion stabilizers, the following should be considered. The presence of the capping agent on the surface should significantly reduce the adsorption energies in comparison to “naked” NPs as the layer of ligands is usually less dense than the bulk metal, and thus, it increases the distance between the residues and the AgNP surface.^104^ The attractive Van der Waals terms in the overall interaction energy are very short-ranged, and thus, they are extremely sensitive to such changes in distances. This also complicates the direct comparison between the *UA* results and experimentally measured adsorption energies.

**Figure 6:**
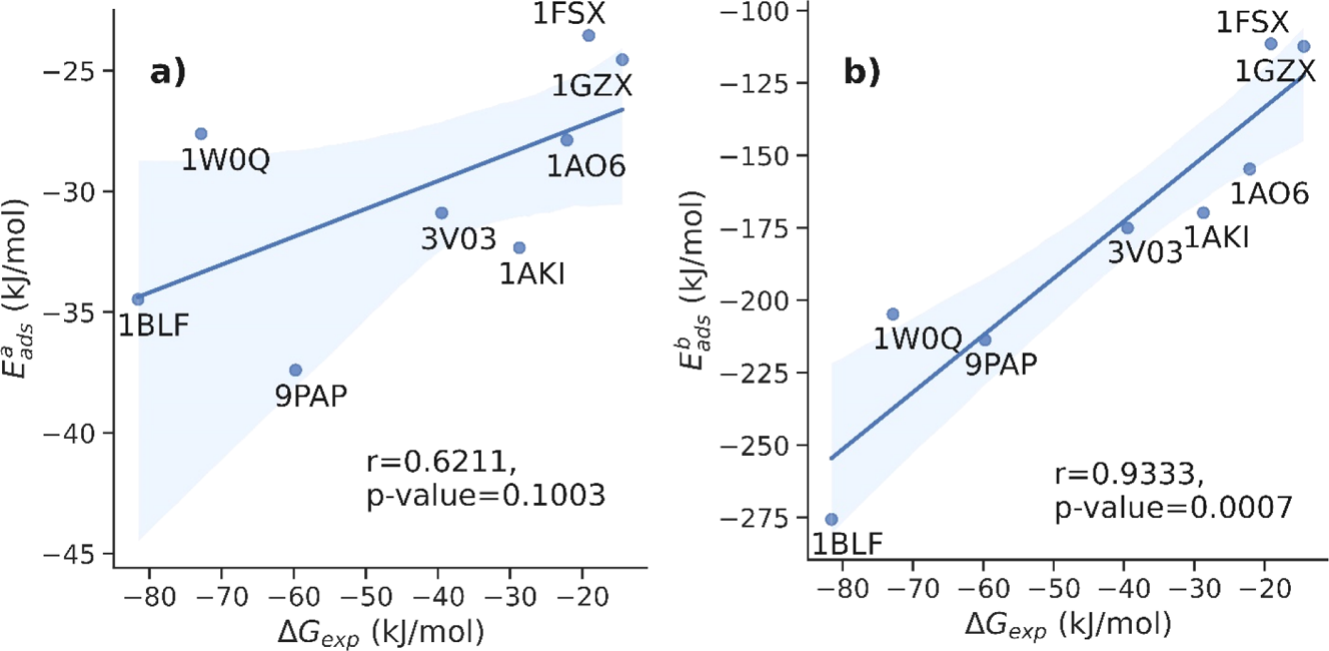
Correlation between the experimentally measured free energy of adsorption Δ*G*_ads_ and *E*_ads_ predicted by the *UA* method: (a) simple average and (b) “Boltzmann” average.

Despite all mentioned limitations of the *UA* approach, we found a good statistical correlation between experimental Δ*G*_ads_ and predicted ensemble-averaged *E*_ads_^B^ values: the calculated correlation coefficient was *r* = 0.93 (*p* < 0.005, Figure 6). A weaker, statistically less relevant correlation (*r* = 0.62, *p* > 0.005) was calculated for *E*_ads_^A^ values. As a result, we propose that the *E*_ads_, calculated with the canonical Boltzmann average approximation (*E*_ads_^B^), can be taken as a better interfacial descriptor for nanoQSAR models, although further investigations are required to validate the statistical performance of this descriptor with a larger dataset of proteins (e.g., by predicting the corona composition^105^).

Previously, it was also proposed to use the adsorption affinity ranking^31^ as a predictive interfacial descriptor of protein adsorption for *in silico* protein corona composition predictions instead of *E*_ads_. For the present set of proteins, a very good match between experimental and calculated adsorption affinity rankings was observed for values obtained with the ensemble average (Table 3). The correct predicted ranking may be the main advantage of the *UA* approach as it allows to model the NP’s biological activity via NanoQSARs.^106^ It has been previously demonstrated that the statistics of the NP protein corona (e.g., weighted relative counts of AA types in the adsorbed proteins) can be quantitatively related to the association of gold or silver NPs with cells.^107^ A relative abundance of the proteins in the corona for a specific material reflects the importance of different contributions to the adsorption energy and should be less sensitive to the absolute values of energy but more sensitive to the affinity ranking.

**Table 3:** Comparison of Binding Affinity Ranking Obtained by Two Averaging Approximations for Adsorption of Proteins Onto Silver NPs Ordered by the Binding Strength

ranking	experimental	simple average approximation	canonical average approximation
1	1BLF	9PAP	1BLF
2	1W0Q	1BLF	9PAP
3	9PAP	1AKI	1W0Q
4	3V03	3V03	3V03
5	1AKI	1AO6	1AKI
6	1AO6	1W0Q	1AO6
7	1FSX	1GZX	1GZX
8	1GZX	1FSX	1FSX

Experimental adsorption energy ranking for selected proteins suggests that glycoproteins (bromelain and bovine lactoferrin) have the highest affinity toward AgNPs. A similar trend for glycoproteins is also confirmed by affinity ranking obtained by the canonical average *UA* approximation. In line with this trend, our calculations have shown that glycosylation should improve overall adsorption on AgNPs, yet the direct contact with the surface is not necessarily maintained through the carbohydrate moiety (Figure S6e,h in Supporting Information).

## Conclusions

In this work, we presented the results of multiscale modeling of adsorption of biomolecules on zero-valent AgNPs using the all-atom MD and the *UA* algorithm. The low computational cost of the *UA* method for predicting protein adsorption energies makes this approach relevant for high-throughput *in silico* probing of binding affinities to inorganic NMs for large datasets of proteins (including glycoproteins and lipoproteins). The *UA* method can be applied for computational prescreening of biomolecules in the development of bioassays and drug nanocarriers for predicting NP protein corona composition or for evaluation of nanotoxicity. The method provides not only the energy of adsorption, calculated as a function of the NP size, shape, and ζ-potential, but it is also capable of predicting the specific three-dimensional structure of NP–protein complexes (nanodocking). The current distribution of the software^108^ is parameterized to predict the energy of physisorption for the range of metal oxides (TiO_2_, SiO_2_, and Fe_2_O_3_), metallic surfaces (Au and Ag), organic NPs (carbon nanotubes, carbon black, and graphene), and semiconductors (CdSe) and can be accessed through NanoCommons Knowledge Base.^109^ The presented multiscale methodology can be further extended to evaluate the adsorption energies at various pH regimes and salt concentrations, which will broaden its applicability for the pharmaceutical and food industries.

## Supporting Information Available

Free energy of adsorption for carbohydrates, lipid fragments, and amino acid side chains; in silico descriptors of protein adsorption at the bio-nano interface; short-range surface adsorption potentials of lipid fragments and carbohydrates onto AgNPs obtained with AWT-MetaD simulations; convergence criteria for PMF corresponding to adsorption of Arg residue onto the Ag(110) surface; water density profiles for silver slabs; orientation of water molecules above the Ag slab surface; adsorption heat maps for selected proteins; lowest energy conformations of adsorption complexes at the Ag(110) surface for selected proteins; and case studies (8) on the adsorption of individual proteins mentioned in the main text (BSA, HSA, BHb, HHb, bromelain, papain, hen egg-white lysozyme, and BLf) (PDF)
