# Supplementary material for: Multiscale Modeling of Bio-Nano Interactions of Zero-Valent Silver Nanoparticles
Source: J Phys Chem B. 2022 Feb 8;126(6):1301–14. doi: 10.1021/acs.jpcb.1c09525 (PMC8859825; doi:10.1021/acs.jpcb.1c09525)
Supplement: Supplementary file 1 — jp1c09525_si_001.pdf [file jp1c09525_si_001.pdf]

Supporting Information

Multiscale modelling of bio-nano interactions of zero-valent silver nanoparticles.

Julia Subbotina, Vladimir Lobaskin

School of Physics, University College Dublin, Belfield, Dublin 4, Ireland

Table of Contents

Tables. 2

Table S1. Free energy of adsorption ΔF_{ads} for carbohydrates, lipid fragments, and amino acid side chains, calculated by numerical integration of PMF curves. 2

Table S2. In silico descriptors of protein adsorption at bio-nano interface (the United Atom method). 3

Figures. 4

Figure S1. Short-range surface adsorption potentials of lipid fragments and carbohydrates onto AgNPs obtained with AWT-MetaD simulations. 4

Figure S2. Convergence criteria for PMF corresponding to adsorption of Arg residue onto Ag(110) surface. 5

Figure S3. Water density profiles for silver slabs. 6

Figure S4. The orientation of water molecules above Ag slab surface. 7

Figure S5. Adsorption heat maps for selected proteins. 8

Figure 6. Lowest energy conformations of adsorption complexes at Ag(110) surface for selected proteins. . 13

Validation Cases. 14

Case 1. Bovine serum albumin (BSA). 14

Case 2. Human serum albumin (HSA). 15

Case 3. Bovine hemoglobin (BHb). 16

Case 4. Human hemoglobin (HHb). 18

Cases 5 and 6. Papain and steam bromelain. 19

Case 7. Hen egg-white lysozyme. 20

Case 8. Bovine lactoferrin (BLf). 21

Bibliography. 22

Tables.

Table S1. Free energy of adsorption ΔF_{ads} for carbohydrates, lipid fragments, and amino acid side chains, calculated by numerical integration of PMF curves.

CG building blocks	Class[a]	T-group[b]	ΔF_{ads}, k_BT		
			**Ag(100)**	**Ag(110)**	**Ag(111)**
(a) Amino acids side chains					
ALA	H	CH_3	0.40	-0.02	0.18
ARG	C^+	NH_2	-8.13	-16.06	-20.61
ASN	P	NH_2	-1.43	-5.25	-5.53
ASP	C^-	O	-0.11	-3.35	-2.23
ASPP	P	COOH	0.09	-5.51	-5.06
CYS	P	SH	-0.77	-4.83	-3.75
CYM	C^-	S	-3.97	-7.92	-5.12
GLN	P	NH_2	-1.80	-6.85	-6.24
GLU	C^-	O	-0.11	-5.90	-3.06
GLUP	P	COOH	-1.57	-6.57	-7.37
GLY[c]	P	CH_2	0.40	-0.02	0.18
HSE/HSD	A	NH	-1.92	-7.63	-8.32
HSP	C^+	NH	-2.34	-6.15	-9.84
ILE	H	CH_3	0.14	-4.67	-3.33
LEU	H	CH_3	-0.05	-8.37	-1.87
LYS	C^+	NH_2	-0.25	-9.89	-3.26
MET	H	CH_3	-3.34	-6.49	-7.97
PHE	A	CH	-3.27	-2.53	-9.93
PRO	P	CH_2	0.24	-1.22	-1.65
SER	P	OH	0.22	-2.08	-0.47
THR	P	OH	0.24	-16.74	-1.90
TRP	A	NH	-9.37	-8.62	-21.09
TYR	A	OH	-10.23	-3.36	-14.83
VAL	H	CH_3	0.34	-0.02	-1.41
(b) Sugars					
AFUC	P	OH	-1.81	-11.35	-13.94
AMAN	P	OH	-2.67	-13.38	-17.20
BGLCNA	P	OH	-15.71	-13.49	-19.63
BGLC	P	OH	-7.08	-11.73	-21.11
BGALNA	P	OH	-19.46	-25.66	-27.40
(c) Lipid fragments					
MAS	P	O	-3.14	-6.50	-5.53
NC4	C^+	CH_3	-0.63	-5.48	-4.93
DMEP	C^-	O	-11.23	-11.32	-14.15
MAMM			0.11	-2.75	-0.28

[a] *H* = hydrophobic, *P* = polar, *A* = aromatic, and *C* = charged

[b] Terminating group in SCA

[c] Same as for ALA

Table S2. In silico descriptors of protein adsorption at bio-nano interface (the *United Atom* method): $\mathbf{E_{ads}^{A,B}}$ – *UA* adsorption energies, $\mathbf{E_{min}}$ - *UA* energy of deepest adsorption minimum, $\mathbf{\Phi_{min}}$, $\mathbf{\Theta_{min}}$, $\mathbf{SSD_{min}}$- *UA* coordinates of deepest adsorption minimum (see Figure S5). **ζ**-potential and radius **R(NP)** are experimentally measured parameters.

PDB ID	Miller index	ζ-potential, mV	R(NP), nm	E_{ads}^{A}, k_BT	E_{ads}^{B}, k_BT	E_{min}, k_BT	Φ_{min}	Θ_{min}	SSD_{min}, nm
3V03	(100)			-7.74	-37.50	-38.21	265.0	100.0	0.232
	(110)	−6.0	40.0	-17.36	-98.67	-99.31	155.0	10.0	0.036
	(111)			-12.28	-75.62	-75.82	160.0	110.0	0.066
	(100)			-7.23	-35.20	-36.29	265.0	100.0	0.282
	(110)	−25.3	60.0	-17.34	-106.47	-107.16	150.0	10.0	0.059
	(111)			-11.88	-71.69	-72.31	150.0	10.0	0.144
1AO6	(100)			-6.36	-33.92	-34.80	355.0	125.0	0.327
	(110)	−6.0	43.0	-16.23	-90.16	-90.64	50.0	25.0	0.004
	(111)			-11.14	-63.13	-64.06	50.0	25.0	0.069
	(100)			-5.95	-29.77	-30.98	40.0	20.0	0.240
	(110)	−14.8	29.2	-15.06	-79.87	-80.22	200.0	115.0	0.017
	(111)			-10.57	-65.39	-65.45	50.0	25.0	0.016
1FSX	(100)			-5.21	-19.14	-20.93	70.0	100.0	0.131
	(110)	−12.5	10.0	-14.53	-72.96	-73.77	345.0	15.0	0.112
	(111)			-8.75	-42.79	-42.88	120.0	70.0	0.187
1GZX	(100)			-5.60	-18.57	-20.19	80.0	55.0	0.290
	(110)	−15.5	15.0	-15.04	-74.09	-74.75	100.0	40.0	0.125
	(111)			-9.06	-43.31	-43.96	100.0	40.0	0.213
1W0Q	(100)			-6.11	-36.26	-36.62	265.0	75.0	0.285
	(110)	−6.0	40.0	-17.11	-118.40	-118.40	265.0	75.0	0.183
	(111)			-10.19	-93.16	-93.21	265.0	80.0	0.212
9PAP	(100)			-9.49	-64.97	-64.97	150.0	95.0	0.267
	(110)	−6.0	40.0	-20.03	-90.37	-90.63	150.0	95.0	0.224
	(111)			-15.74	-103.29	-103.61	155.0	45.0	0.239
1AKI	(100)			-6.17	-35.70	-36.49	225.0	125.0	0.263
	(110)	−6.0	40.0	-18.42	-90.72	-91.03	245.0	120.0	0.164
	(111)			-14.54	-79.06	-79.71	230.0	130.0	0.201
1BLF	(100)			-8.09	-49.02	-49.46	115.0	50.0	0.234
	(110)	−28.1	18.0	-19.79	-145.04	-145.30	100.0	55.0	0.085
	(111)			-13.83	-139.50	-139.75	100.0	55.0	0.117

Figures.

Figure S1. Short-range surface adsorption potentials of lipid fragments and carbohydrates onto AgNPs obtained with AWT-MetaD simulations.
Vertical dashed lines correspond to the positions of maxima in density profiles for water molecules and counter ions around the metallic slab. (a) The FCC (100) surface. (b) The FCC (110) surface. (c) The FCC (111) surface.

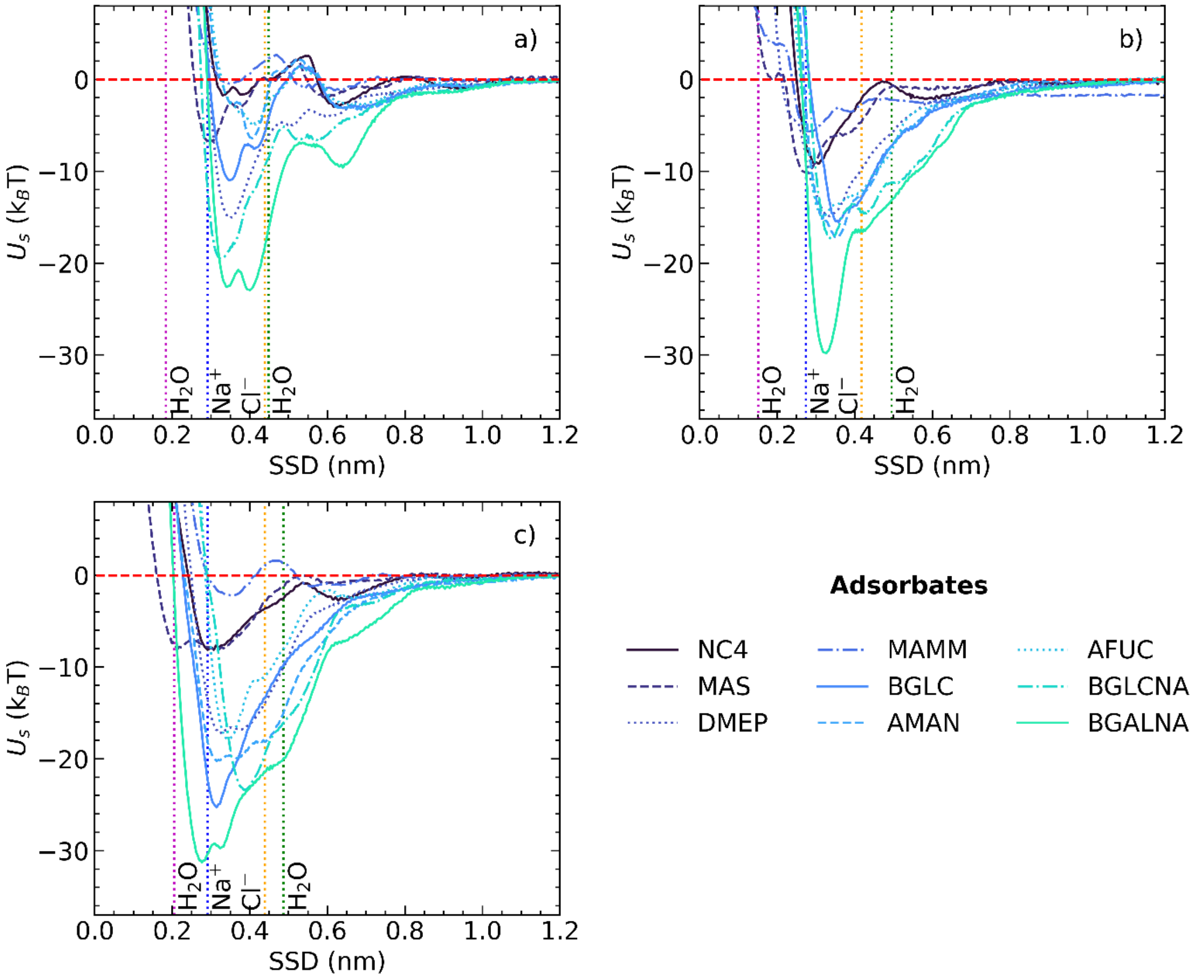

Figure S2. Convergence criteria for PMF corresponding to adsorption of Arg residue onto Ag(110) surface.

(a) Time evolution of collective variable SSD during AWT-Metadynamics. (b)Time evolution of hill heights during AWT-Metadynamics. (c) Time evolution of energy differences between global minimum (state A) and other locate minima (states B-D). Over the time (> 400 ns) the energy difference become constant.

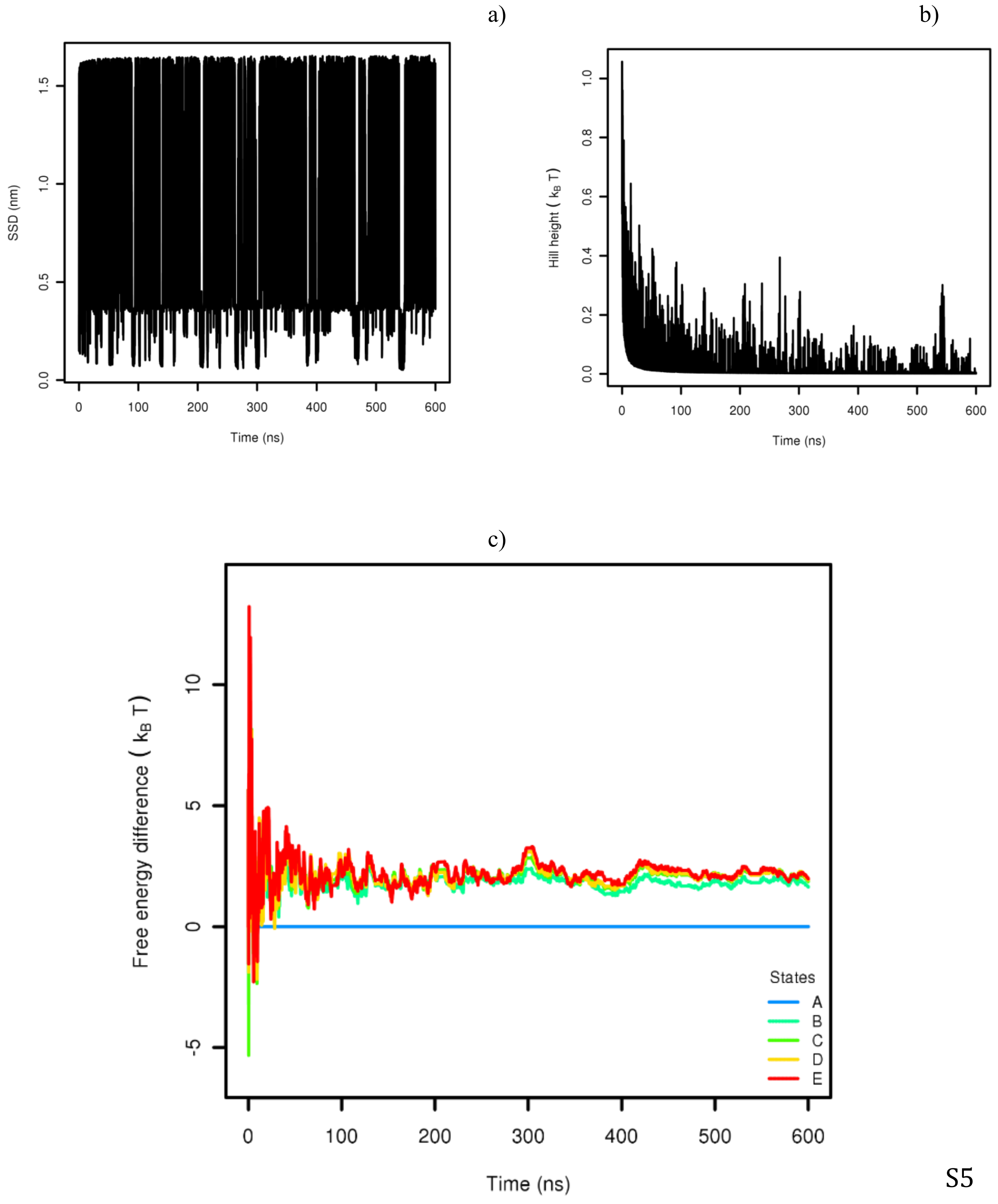

Figure S3. Water density profiles for silver slabs. Two peaks at 0.15-0.20 nm and 0.45-0.49 nm distance away from the surface were located: (a) Ag(100), (b) Ag(110), (c) Ag(111).

a)

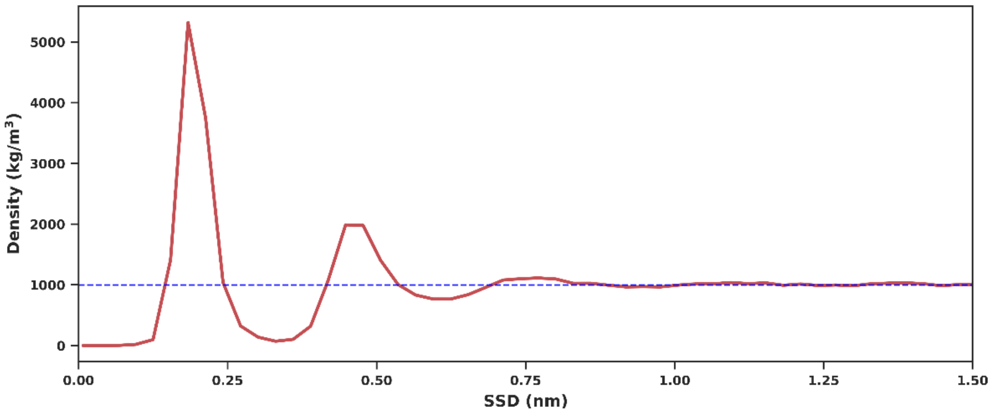

b)

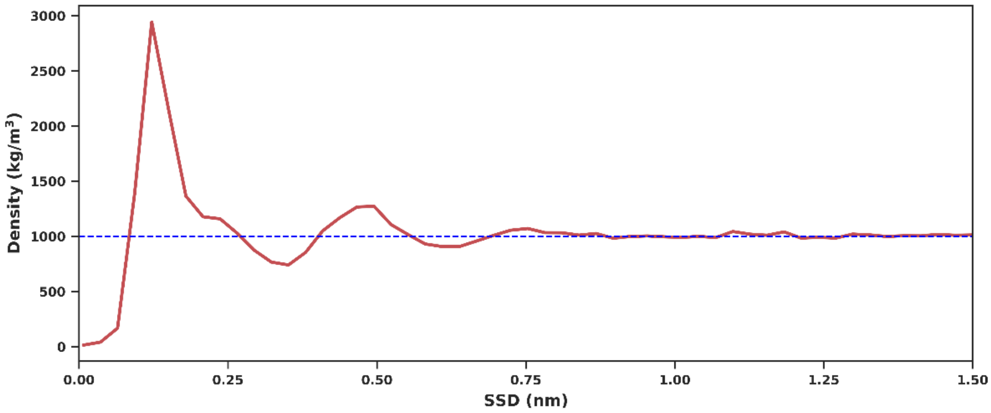

c)

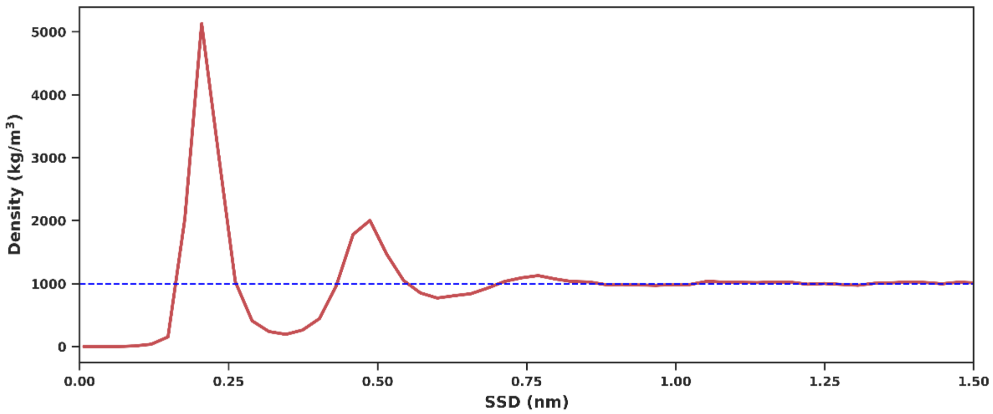

Figure S4. The orientation of water molecules above Ag slab surface.
β is the angle between the dipole moment of water and the Z-axis of the box, which is a norm to the slab surface: (a) Ag(100), (b) Ag(110), (c) Ag(111).

a)

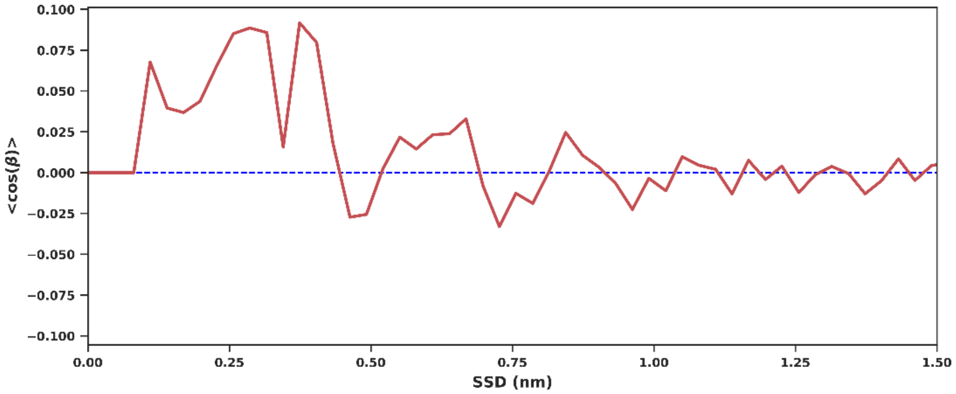

b)

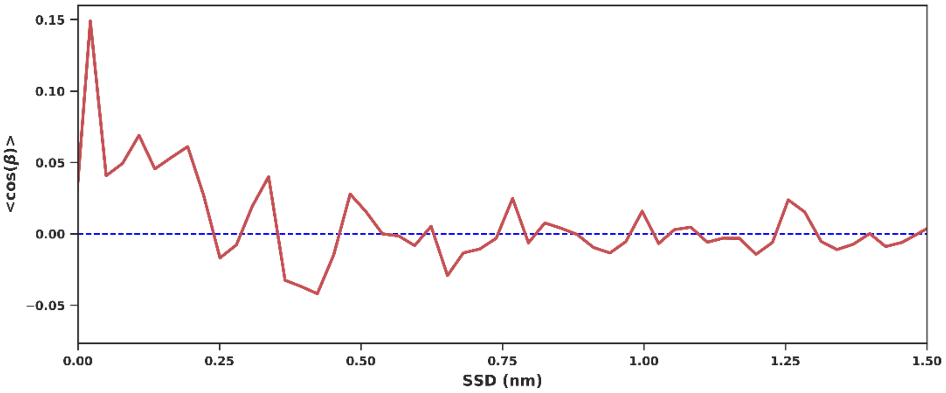

c)

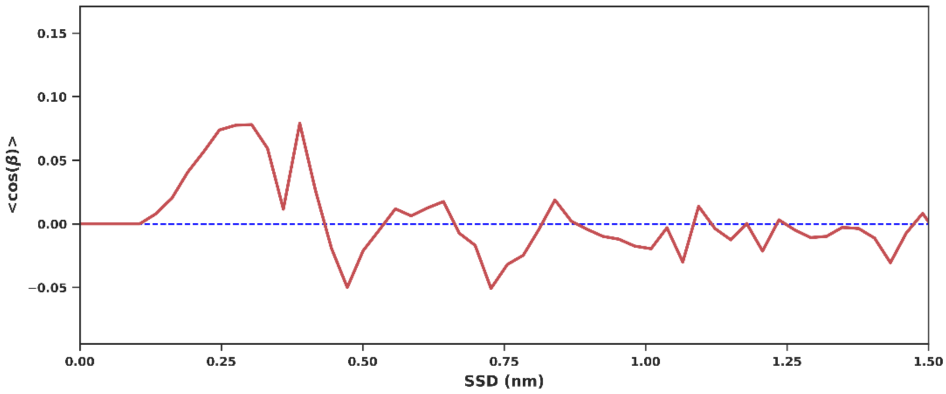

Figure S5. Adsorption heat maps for the adsorption of proteins from cases 1-8 onto silver nanoparticles.

a) BSA(PDB ID:3V03)

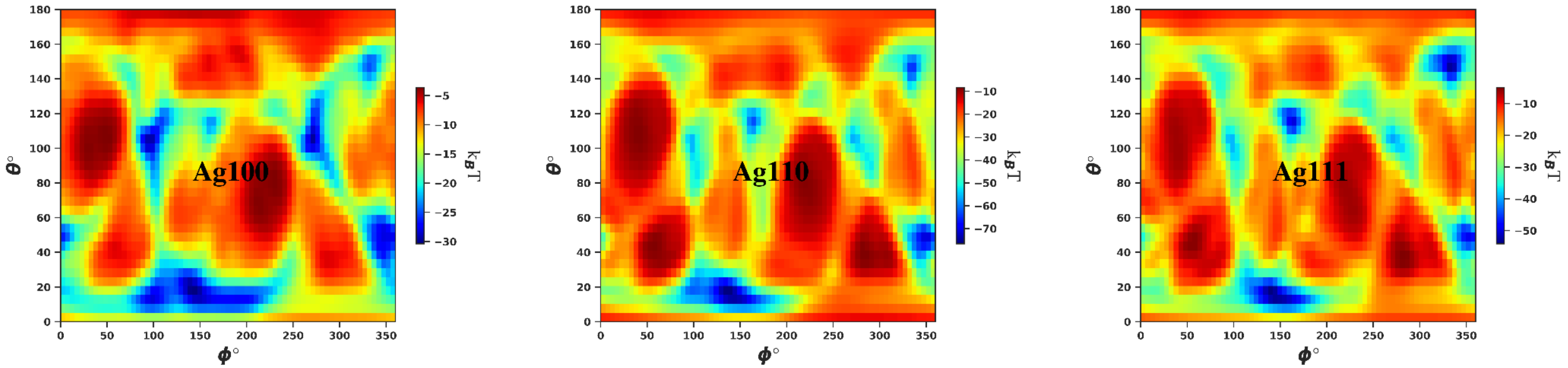

b) HSA at 29.2 nm AgNP (PDB ID: 1AO6)

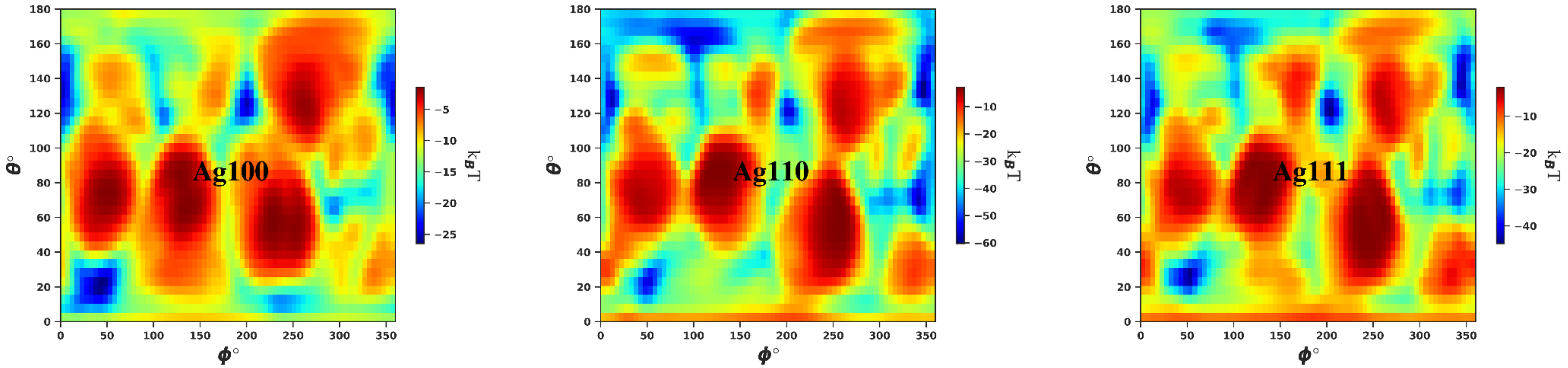

c) HSA at 43.0 nm AgNP (PDB ID: 1AO6)

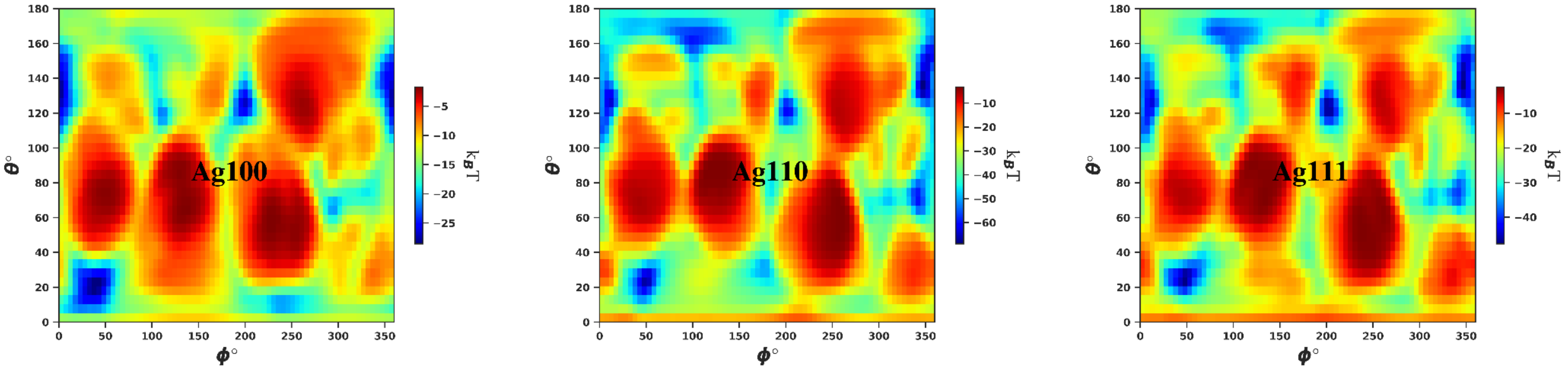

d) BHb (PDB ID: 1FSX)

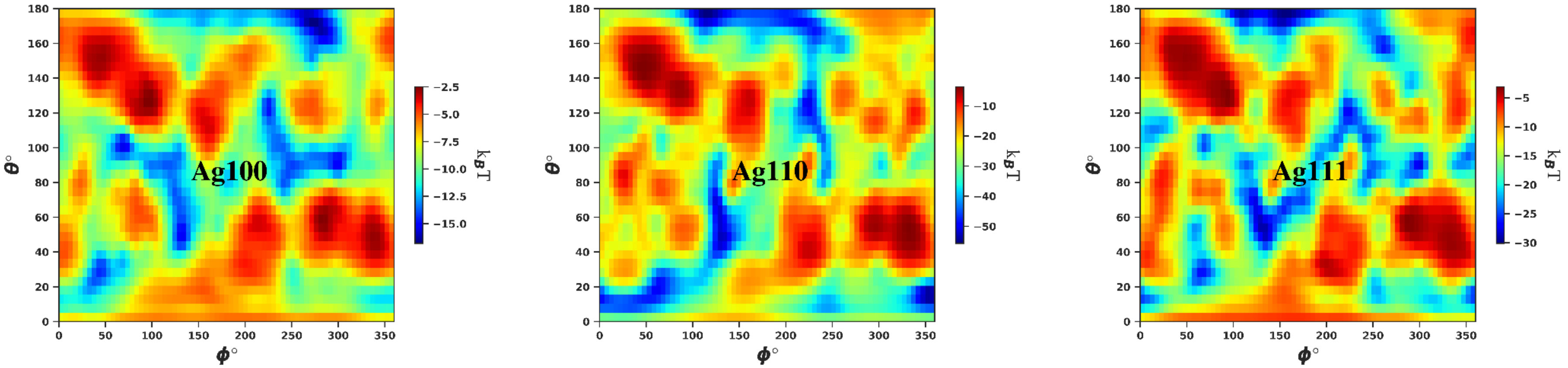

e) HHb (PDB ID: 1GZX)

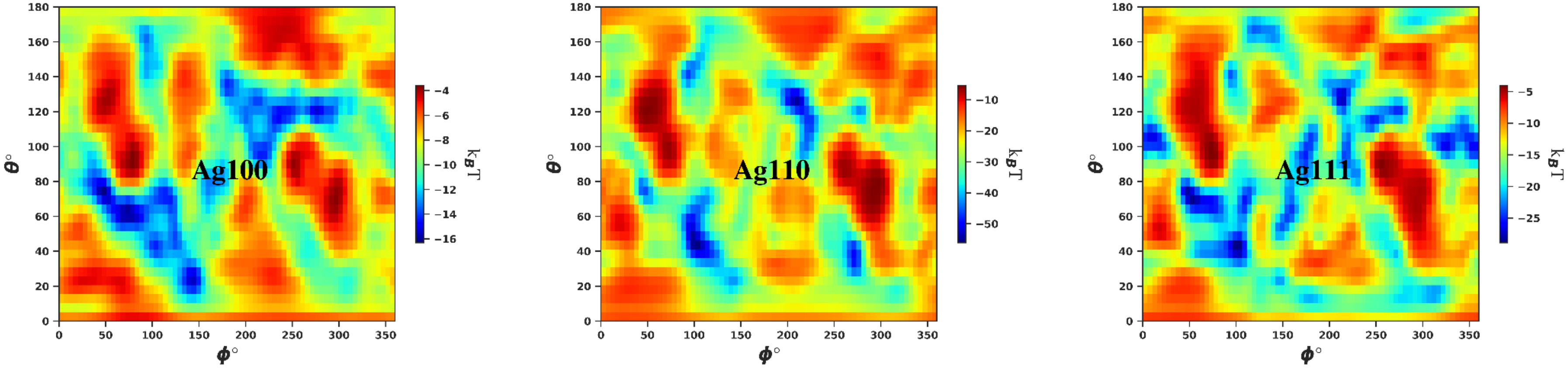

f) Bromelain (PDB ID: 1W0Q)

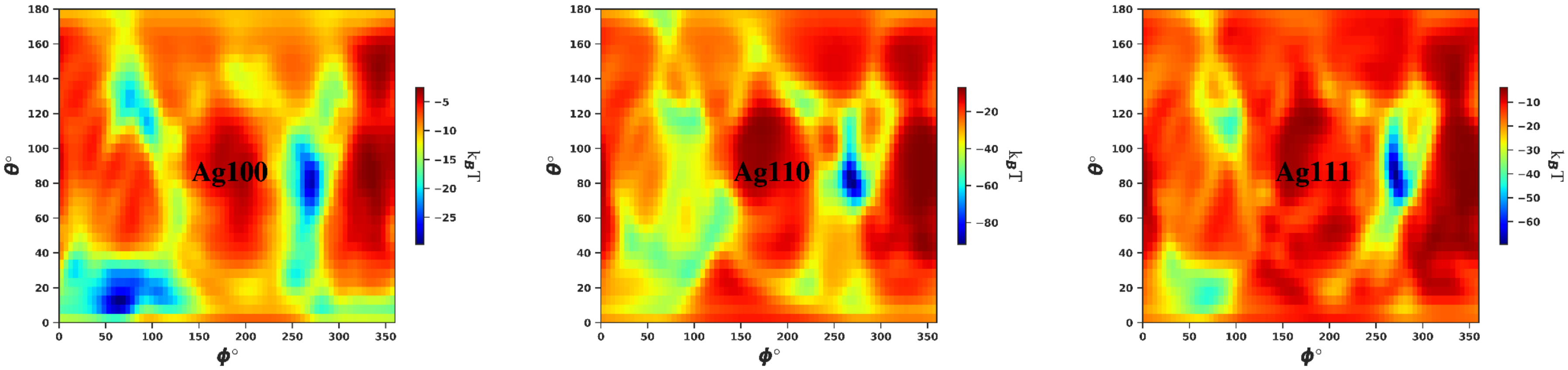

g) Papain (PDB ID: 9PAP)

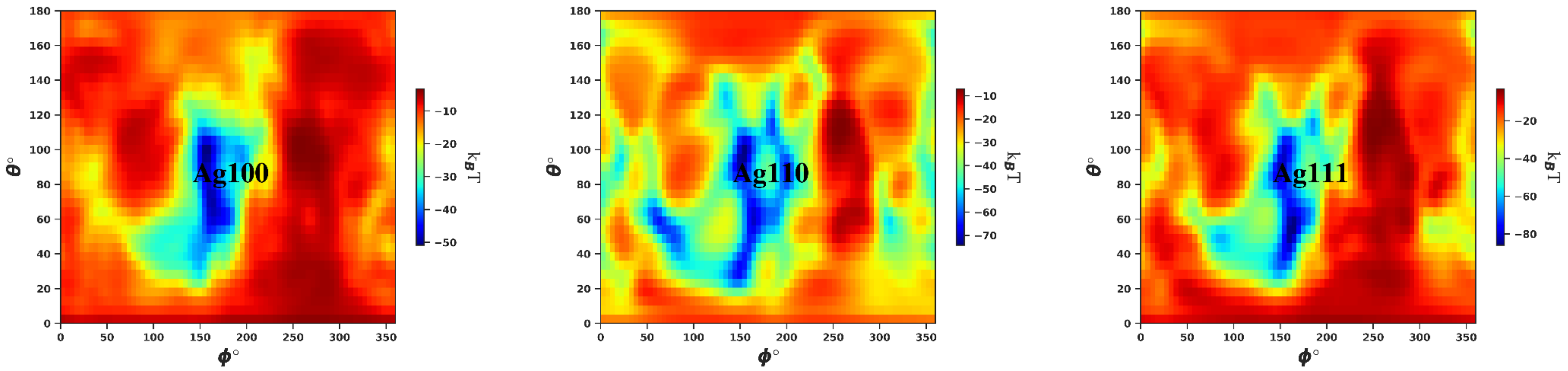

h) Hen egg-white lysozyme (PDB ID: 1AKI)

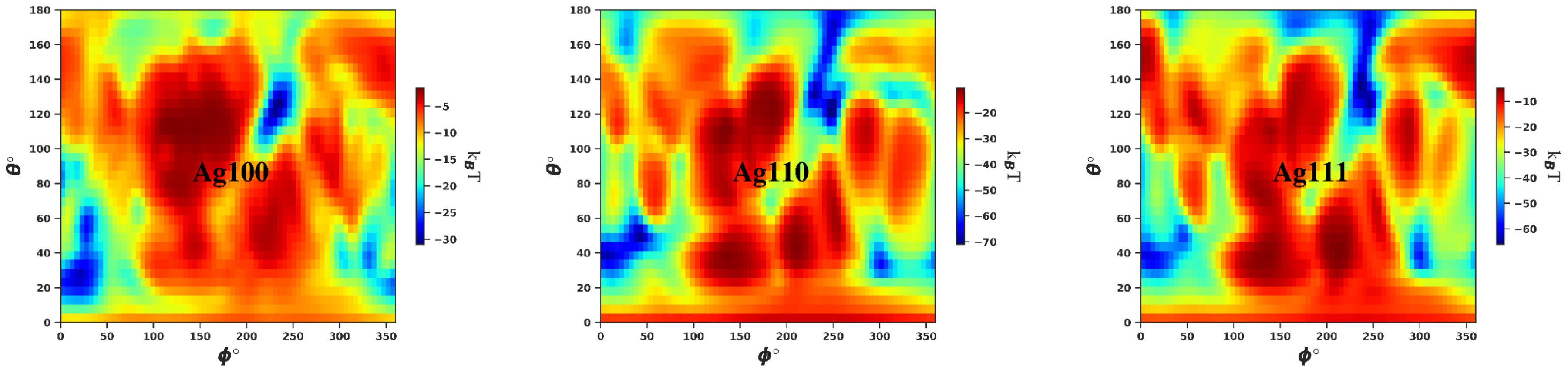

i) BLf (PDB ID: 1BLF)

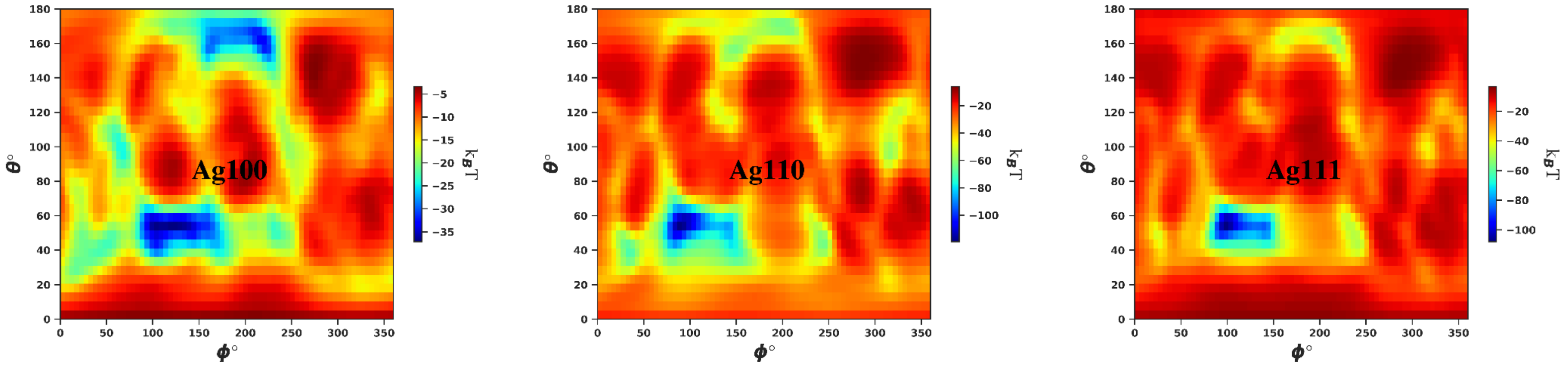

Figure 6. Lowest energy conformations of adsorption complexes at Ag(110) surface for selected proteins: BSA (a), HSA (b), BHb (c), HHb (d), bromelain (e), papain (f), lysozyme (g), and BLf (h).

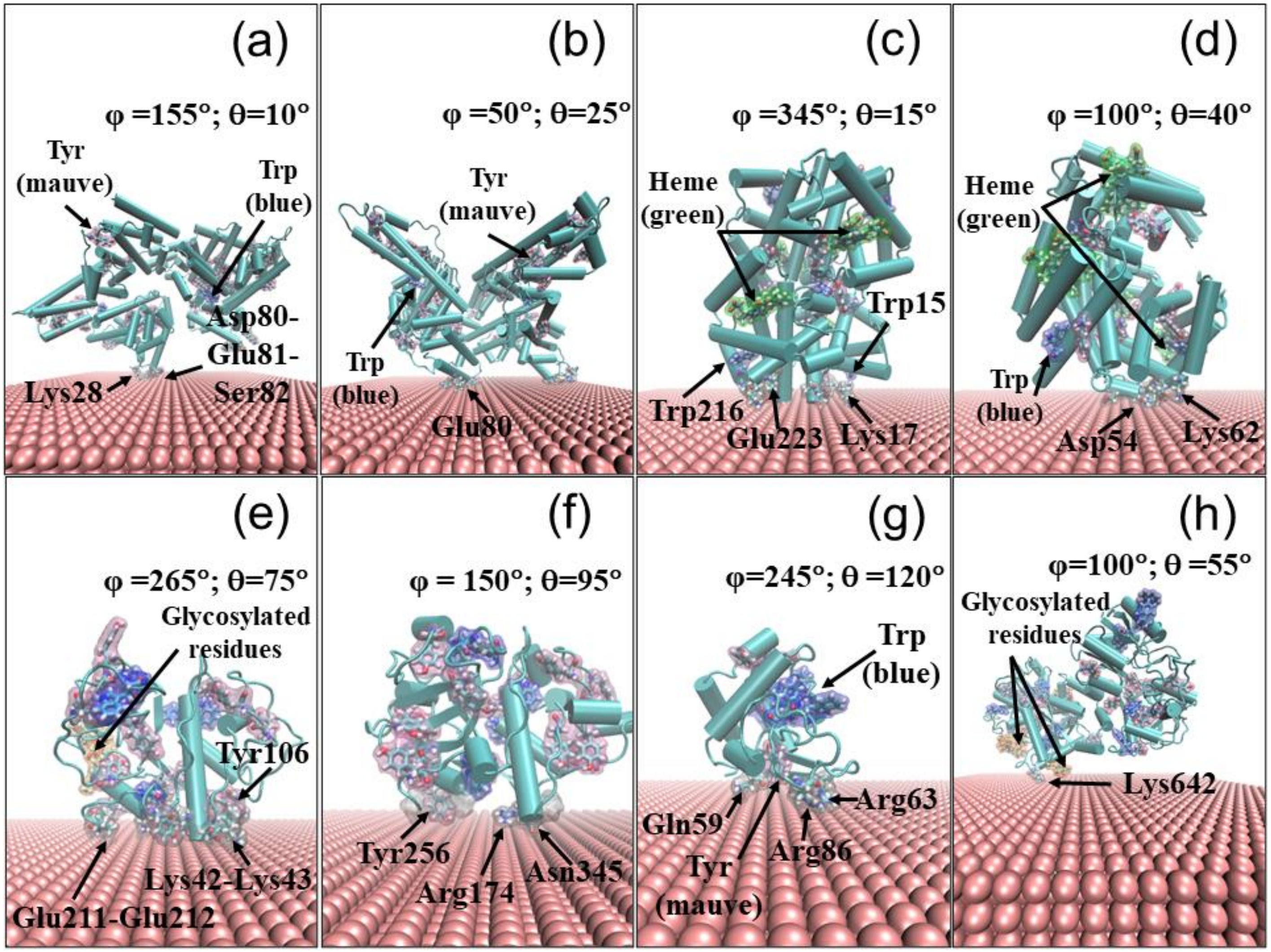

Validation Cases.

Case 1. Bovine serum albumin (BSA).

The adsorption of BSA protein (PDBID: 3V03) was studied experimentally by various methods[1–4]. According to the SFS results[5], BSA adsorption onto 40 nm AgNPs altered the micro-environment of Tyr amino acids in the protein, decreasing their hydrophilicity. At the same time, almost no change was observed for the emission peaks of the tryptophan (Trp) residues upon binding, suggesting that no changes occurred in their micro-environment. The decrease of fluorescence intensity measured for Trp158 (on the BSA surface) and Trp237 (interior part of BSA) upon addition of 60 nm AgNPs[6] was associated with the quenching effect of AgNPs only. The interaction between nanoparticles with BSA occurred via the binding site "proximal" to tryptophan residues. In both studies[5,6], the structural changes in BSA were not substantial according to CD spectra. The estimated α-helicity decreased from 56.21% to 49.85% for 40 nm AgNPs, while the interaction of BSA with 60 nm AgNPs led to a change in protein's α-helical content from 67.4% to 64.7%.

AgNPs used in both studies were spherical and had a similar face-centred cubic (FCC) morphology with (111), (220), (200) facets. The ζ-potential reported for 60-nm AgNP was in the range of −25.3 mV to –51.9 mV[7]. The ζ-potential for 40-nm AgNPs was not measured in original paper[5], but another source[8] reported ζ-potential of −6 mV for the distribution of 40-70 nm spherical AgNPs synthesized in presence of poly(*N*-vinylpyrrolidone) (PVP). The authors mentioned, that selected polymeric shell on AgNPs is expected to cause a minimal effect on their properties. Keeping this assumption in the mind, the reported value of ζ-potential was chosen to model protein adsorption on bare 40 nm AgNP by *UA* approach. The experimentally measured free energy of adsorption ΔG_{ads} for BSA on AgNP (40nm) was −39.49 kJ/mol[5]. No thermodynamic parameters were not reported[6] for interaction with 60nm AgNPs.

Using these values, the predicted adsorption energy E_{ads}^{B} for 40 nm AgNPs averaged across all facets, was -70.60 k_BT (Table S2) or -175.01 kJ/mol (Table 2). The strongest binding was predicted for Ag(110) surface, followed by binding to Ag(111) facet. The preferred orientation of BSA above Ag surface was located at rotational angles φ =155° and θ =10° (Figure S5a) and was similar across all facets. The spatial arrangement for BSA-Ag(110) complex is shown in Figure 6a of the main text. Lys28 and Asp80-Glu81-Ser82-His83-Ala84 fragments were in proximity of the silver surface. Tryptophan residues were found relatively far away from the surface.

Case 2. Human serum albumin (HSA).

HSA protein (PDBID:1AO6) is almost 76.6% identical to BSA (Figure S7). The observed adsorption behaviour of HSA on triangle[9] and spherical[10] AgNPs was similar to that of BSA. After adsorption, the loss of α-helicity by HSA was confirmed via CD experiments.
This change was relatively small, ~6% (triangle NPs) and about 4% (spherical NPs), and sensitive to the concentration of added NPs. UV–Vis absorption spectra supported the hypothesis of the interaction of Trp and Tyr residues with triangle AgNPs through electrostatic forces[9]. SFS confirmed the conformational change of microregion near Tyr and Trp residues after HSA binding to spherical AgNPs, which resulted in their increased hydrophilicity[10]. Measured ζ-potential for spherical (29.2 nm) silver NPs was −14.8 mV; no ζ-potential value was reported for the triangle (43.0 nm) NPs. Free energy of HSA adsorption measured for triangle NPs was slightly lower (−22.14 kJ/mol[10]) than ΔG_{ads} for bovine albumin in previous case. No ΔG_{ads} value was reported for spherical NPs used in study.

Figure S7. Sequence alignment between BSA and HSA proteins.

```
P02769 ALBU_BOVIN        1   MKWVTFISLLLLFSSAYSRGVFRRDTHKSEIAHRFKDLGEEHFKGLVLIAFSQYLQQCPF    60
P02768 ALBU_HUMAN        1   MKWVTFISLLFLFSSAYSRGVFRRDAHKSEVAHRFKDLGEENFKALVLIAFAQYLQQCPF    60
                             **********:**************:****:**********:**.******:********

P02769 ALBU_BOVIN       61   DEHVKLVNELTEFAKTCVADESHAGCEKSLHTLFGDELCKVASLRETYGDMADCCEKQEP   120
P02768 ALBU_HUMAN       61   EDHVKLVNEVTEFAKTCVADESAENCDKSLHTLFGDKLCTVATLRETYGEMADCCAKQEP   120
                             ::*******:************   .*:*********:**.**:******:***** ****

P02769 ALBU_BOVIN      121   ERNECFLSHKDDSPDLPKL-KPDPNTLCDEFKADEKKFWGKYLYEIARRHPYFYAPELLY   179
P02768 ALBU_HUMAN      121   ERNECFLQHKDDNPNLPRLVRPEVDVMCTAFHDNEETFLKKYLYEIARRHPYFYAPELLF   180
                             *******.****.*:**:*  :*: :.:*   *: :*:.*  ********************:

P02769 ALBU_BOVIN      180   YANKYNGVFQECCQAEDKGACLLPKIETMREKVLASSARQRLRCASIQKFGERALKAWSV   239
P02768 ALBU_HUMAN      181   FAKRYKAAFTECCQAADKAACLLPKLDELRDEGKASSAKQRLKCASLQKFGERAFKAWAV   240
                             :*::*:..* ***** **.******:: :*::   ****:***:***:*******:***:*

P02769 ALBU_BOVIN      240   ARLSQKFPKAEFVEVTKLVTDLTKVHKECCHGDLLECADDRADLAKYICDNQDTISSKLK   299
P02768 ALBU_HUMAN      241   ARLSQRFPKAEFAEVSKLVTDLTKVHTECCHGDLLECADDRADLAKYICENQDSISSKLK   300
                             *****:******.**:**********.*********************:***:******

P02769 ALBU_BOVIN      300   ECCDKPLLEKSHCIAEVEKDAIPENLPPLTADFAEDKDVCKNYQEAKDAFLGSFLYEYSR   359
P02768 ALBU_HUMAN      301   ECCEKPLLEKSHCIAEVENDEMPADLPSLAADFVESKDVCKNYAEAKDVFLGMFLYEYAR   360
                             ***:**************:* :* :** *:***.*.******* ****.*** *****:*

P02769 ALBU_BOVIN      360   RHPEYAVSVLLRLAKEYEATLEECCAKDDPHACYSTVFDKLKHLVDEPQNLIKQNCDQFE   419
P02768 ALBU_HUMAN      361   RHPDYSVVLLLRLAKTYETTLEKCCAAADPHECYAKVFDEFKPLVEEPQNLIKQNCELFE   420
                             ***:*:* :****** **:***:***  *** **:.***::* **:**********: **

P02769 ALBU_BOVIN      420   KLGEYGFQNALIVRYTRKVPQVSTPTLVEVSRSLGKVGTRCCTKPESERMPCTEDYLSLI   479
P02768 ALBU_HUMAN      421   QLGEYKFQNALLVRYTKKVPQVSTPTLVEVSRNLGKVGSKCCKHPEAKRMPCAEDYLSVV   480
                             :**** *****:****:***************.*****::**.:**::****:*****::

P02769 ALBU_BOVIN      480   LNRLCVLHEKTPVSEKVTKCCTESLVNRRPCFSALTPDETYVPKAFDEKLFTFHADICTL   539
P02768 ALBU_HUMAN      481   LNQLCVLHEKTPVSDRVTKCCTESLVNRRPCFSALEVDETYVPKEFNAETFTFHADICTL   540
                             **:***********::*******************  ******* *: : **********

P02769 ALBU_BOVIN      540   PDTEKQIKKQTALVELLKHKPKATEEQLKTVMENFVAFVDKCCAADDKEACFAVEGPKLV   599
P02768 ALBU_HUMAN      541   SEKERQIKKQTALVELVKHKPKATKEQLKAVMDDFAAFVEKCCKADDKETCFAEEGKKLV   600
                              :.*:***********:*******:****:**::*.***:*** *****:*** ** ***

P02769 ALBU_BOVIN      600   VSTQTALA-                                                      607
P02768 ALBU_HUMAN      601   AASQAALGL                                                      609
                             .::*:**.
```

The current version of the *UA* model includes corrections for adsorption energy accounting for a shape of NPs (cube, rode, sphere). Corrections for triangle nanoparticles were not developed yet. To model HSA adsorption onto triangle NP, a spherical NP model was applied (Table S2). Obtained value of E^{B}_{ads} was -62.4 k_BT (-154.70 kJ/mol). The lowest energy conformation of the HSA-AgNP complex was predicted at rotational angles φ =50° and θ =25° (Figure S5c and Figure 6b in the main text). In this orientation, the closest to silver surface residues were located on α-helices at Asp79-Glu80 and in the loop at Lys587. Residues Thr589, Glu593, Lys596-Lys597 were also in proximity of the slab.

Case 3. Bovine haemoglobin (BHb).

Interaction between bovine hemoglobin (PDBID: 1FSX) and AgNP (5–10 nm) was previously studied by fluorescence, UV–Vis, and CD spectroscopy at the range of

temperatures[11]. Demonstrated dynamic quenching at fluorescence spectra suggested that the interaction between NP and BHb occurs via the single binding site. The appearance of the Soret band in UV-Vis absorption spectra indicated that AgNPs directly attack and degrade heme fragments. Fluorescent quenching data also suggested that tryptophan residues act as a binding site for silver NP, or they are located very close to it.

Thermodynamic parameters measured during this study were as follows: ΔG =−19.1 kJ/mol, ΔH = 189.85 kJ/mol, and ΔS = 208.95 J mol^{-1} K^{-1}at 293.15 K. That suggests, that binding of AgNPs to BHb occurs spontaneously (ΔG <0), the binding process is entropy-driven and endothermic (ΔH>0, ΔS>0).

Results of CD spectroscopic studies[11] have shown an approximately 50% decrease of α-helix and a 20% increase of β-folds. The alpha-beta transition caused by the addition of silver nanoparticles enhanced protein hydrophobicity. That is in line with thermodynamic properties indicating that binding is associated with the hydrophobic effect.

To model the interaction of BHb with AgNP only the polypeptide part of the protein was considered, the heme fragment was excluded from the consideration due to the lack of corresponding short-range surface potential $U_{i,s}^{\mathrm{nb}}\left(h_i(d_i,\theta,\phi)\right)$ for heme bead. No ζ-potential was reported in the original BHb adsorption experiment. Although, a ζ-potential for the similar size (4–12 nm) distribution of biosynthesized AgNPs was reported in work[12]: measured ζ-potential was -12.5 –(-30.4) mV. The highest value, -12.5 mV, was applied for UA modelling of BHb adsorption on the spherical silver NP (Table S2).

Averaged adsorption energy E_{ads}^{B} calculated for BHb was -44.96 k_BT (-111.46 kJ/mol). The value confirms simultaneous adsorption of the bovine hemoglobin. For the lowest energy conformation of adsorption complex with rotational coordinates φ =345° and θ =15° (Figure S5d) the closest contacting residues were Lys17 in α-subunit, Lys118 and Lys120 in β-subunit, Glu223, and Lys321-Glu322 site at the C-terminus (Figure 6c). The tryptophan

residue Trp15 was found 1.0 nm away from the silver surface. Both heme segments (they were kept in original PDB structure during post factum reconstruction of adsorption complex) would not interact directly with silver atoms (Figure S6c, green-coloured fragments). The closest heme segment was 2.2 nm away from the surface. Adding heme-Ag short-range surface potential $U_{i,s}^{\mathrm{nb}}\left(h_i(d_i, \theta, \phi)\right)$ to the *UA* model might be necessary to improve the prediction for adsorption of heme-containing biomolecules.

Case 4. Human hemoglobin (HHb).

The adsorption of HHb (PDBID: 1GZX) on spherical AgNPs was studied in work[13]. Synthesized AgNPs with a net negative charge had (111), (200), (220), (311), (222) crystal planes present in the FCC phase of the unit cell. The average size of NPs determined by transmission electron microscopic (TEM) analysis was 15 nm. The measured ζ-potential was −15.5 mV. The absorption spectra of the HHb-AgNPs complex showed the change in intensity of tryptophan band, ε band, Soret band and Q-band. This observation points to the interaction of AgNPs with heme moiety and tryptophan residues, similarly to BHb. The electrostatic interaction between positively charged residues and negatively charged AgNPs was also proposed as an attractive force for adsorption. After conjugation with AgNPs and corona formation, HHb retained the helical structure: the percentage of α-helix structures decreased by about 10%, while β-sheet content stayed almost unchanged, ca. 8%. Free energy of adsorption measured in this study was ΔG =−14.428 kJ/mol (298 K), while enthalpic and entropic terms were ΔH =− 99.19 kJ/mol and ΔS =14.794 J mol^{-1} K^{-1}.

The experimental parameters listed above were used to calculate the adsorption energy for HHb. Calculated values for (111), (110), and (100) FCC planes are listed in Table S2. The average value for E_{ads}^{B} was -45.32 k_BT (-112.36 kJ/mol). Human hemoglobin was calculated to have a preference for Ag(110) facet (Table S2). Multiple minima corresponding to

adsorbed orientations of the protein were observed (Figure S5e). The most favourable orientation was located at rotational coordinates φ =100° and θ =40°. In that orientation, the contact between AgNP and hemoglobin occurred at Asp54 and Lys62 on β-subunit (Figure 6d in the main text). Tryptophane and heme fragments were found within 1.5 nm of the surface. Similarly to the previous case, heme-AgNP short-range surface potential $U_{i,s}^{\mathrm{nb}}\left(h_i(d_i,\theta,\phi)\right)$ is necessary to improve the prediction of absorption for hemoglobin proteins.

Cases 5 and 6. Papain and steam bromelain.

Interaction of papain (PDBID: 9PAP) and bromelain (PDBID:1W0Q) with AgNP was shown[14] to be driven by hydrophobic and electrostatic interactions as well. Both proteins belong to the class of cysteine-proteases. Bromelain is known to be a glycoprotein and can serve as a great example for testing the *UA* model to predict adsorption energy for more complex adsorbates, such as glycoproteins and lipoproteins.

The AgNPs had a nearly spherical to elliptical shape and an average size of 40 ± 5 nm. The ζ-potential and the morphology of crystal planes were not reported in the study. Measured free energy of interaction between bromelain and AgNPs was $\Delta G = -72.853$ kJ/mol at 298 K. Reported value for papain was $\Delta G = -59.763$ kJ/mol at 298 K. Enthalpic and entropic terms were $\Delta H =–-39.354$ kJ/mol and $\Delta S = 112.413$ J mol^{-1} K^{-1} for bromelain, and $\Delta H =–-15.679$ kJ/mol and $\Delta S = 150.456$ J mol^{-1} K^{-1} for papain.

UA averaged adsorption energy E_{ads}^{B} (Table S2) for bromelain was -82.61 k_BT (-204.78 kJ/mol) and for papain -86.21 k_BT (-213.71 kJ/mol). The lowest energy conformation for bromelain - Ag(110) complex was found at rotational coordinates φ =265° and θ =75° (Figure S5f). Intriguingly, regardless of calculated strong attractive short-range interaction between carbohydrate fragments and Ag slab, the glycosylated residues of bromelain were

not predicted to interact directly with silver atoms (Figure 6e). Instead, the interaction occurred via Glu211-Glu212 residues at the terminus and via the Lys42-Lys43 fragment. For the papain, binding sites were located at Arg174, Tyr256, and Asn345 residues (Figure 4f).

Case 7. Hen egg-white lysozyme.

Adsorption of hen egg-white lysozyme (PDBID: 1AKI) protein on bare AgNPs was also reported[15]. NPs synthesized in this study showed three distinct diffraction peaks in XRD spectra corresponding to (111), (200), and (220) planes of FCC silver. The average size of prepared spherical NPs was 40±5 nm. Synchronous fluorescence spectroscopy/CD spectra confirmed, that adsorption of lysozyme led to alterations of the microenvironment around tryptophane residues, so they became less easily exposed to the aqueous medium. At the same time, the microenvironment of tyrosine was not impacted by adsorption. Binding to the silver surface caused the decrease of α-helix content of lysozyme from 37.41% to 30.94 – 34.35% depending on the concentration of AgNPs added. Measured free energy of adsorption for this protein was $\Delta G = -28.71$ kJ/mol at 298K, the enthalpy was $\Delta H = 124.68$ kJ/mol, and the entropy was $\Delta S = 514.73$ J mol^{-1} K^{-1}. No ζ-potential for silver NPs was reported in this study. Similarly to the cases described earlier, the value of −6.0 mV was applied for *United Atoms* computations (Table S2).

Calculated averaged E_{ads}^{B} for lysozyme was -68.49 k_BT (-169.79 kJ/mol). The lowest energy orientation for Ag(110) crystal plane was located at rotational angles φ =245° and θ =120° (Figure S5h). The closest contacts between nanomaterial and the protein in this configuration were Gln59, Arg63, and Arg86 (Figure 6g). Aromatic residue Tyr71 was also relatively close to the nano surface (<0.6 nm). Yet, tryptophan residues in this conformation were facing away from the slab.

Case 8. Bovine lactoferrin (BLf).

The formation of bovine lactoferrin BLf (PDBID:1BLF) corona on AgNP was studied in work[16]. Lactoferrin represents the case of glycosylated protein, isolated from secrete fluids such as milk, saliva, and tears. The estimated average diameter of BLf was 7.8 nm, while spherical AgNPs initially had a dimeter of 18±3 nm. The reported ζ-potential for AgNPs was −28.1 mV. The high negative potential was the result of the presence of a citrate capping agent at the NPs surface. Adsorption of BLf was observed to proceed through the formation of a single layer of BLf , which led to the increase of particle diameter up to 20±10 nm. The diffraction peaks of pristine AgNPs corresponded to (111), (200), (222) planes of FCC crystal. The interaction between NPs and lactoferrin was governed by Van der Waals and hydrogen bond interactions, since changes in enthalpy and entropy were negative, ΔH=-4.6±0.21 kJ/mol and ΔS=-15.98 ±1.02 kJ/mol/ºC. The reported value for the free energy of adsorption was ΔG=-81.59 kJ/mol. The CD spectra suggested that bovine lactoferrin remains relatively stable at low concentrations of AgNPs, but at higher concentrations, the α-helical content of protein drops by 10 % (from 25.21% to 16.99%).

Adsorption energy E_{ads}^{B} for bovine lactoferrin calculated with given experimental parameters (Table S2) was the highest among all selected proteins, -111.19 k_BT (275.63 kJ/mol).

The lowest energy orientation was observed at the region with φ =100° and θ =55° (Figure S5i). In that conformation, Ag atoms interact with positively charged Lys642 residue. Iron binding sites in the N-lobe (Asp79, His272, Tyr111) and C-lobe (Arg140, Tyr211) of lactoferrin were not identified as Ag binding sites. Glycosylated residues did not interact directly with the metallic part, but were nearby (> 0.5 nm, Figure 6h in the main text). Aromatic residues Tyr (mauve surface) and Trp (blue surface) were not in immediate contact with the Ag slab as well.

Bibliography.

(1) Iosin, M.; Canpean, V.; Astilean, S. Spectroscopic Studies on PH- and Thermally Induced Conformational Changes of Bovine Serum Albumin Adsorbed onto Gold Nanoparticles. *J. Photochem. Photobiol. A Chem.* **2011**, *217* (2–3), 395–401.

(2) Ravindran, A.; Singh, A.; Raichur, A. M.; Chandrasekaran, N.; Mukherjee, A. Studies on Interaction of Colloidal Ag Nanoparticles with Bovine Serum Albumin (BSA). *Colloids Surfaces B Biointerfaces* **2010**, *76* (1), 32–37.

(3) Gebregeorgis, A.; Bhan, C.; Wilson, O.; Raghavan, D. Characterization of Silver/Bovine Serum Albumin (Ag/BSA) Nanoparticles Structure: Morphological, Compositional, and Interaction Studies. *J. Colloid Interface Sci.* **2013**, *389* (1), 31–41.

(4) Siddiq, A. M.; Murugan, D.; Srivastava, R.; Alam, M. S. Influence of PH on Interaction of Silver Nanoparticles - Protein: Analyses by Spectroscopic and Thermodynamic Ideology. *Colloids Surfaces B Biointerfaces* **2019**, *184*, 110524.

(5) Wang, G.; Lu, Y.; Hou, H.; Liu, Y. Probing the Binding Behavior and Kinetics of Silver Nanoparticles with Bovine Serum Albumin. *RSC Adv.* **2017**, *7* (15), 9393–9401.

(6) Dasgupta, N.; Ranjan, S.; Patra, D.; Srivastava, P.; Kumar, A.; Ramalingam, C. Bovine Serum Albumin Interacts with Silver Nanoparticles with a “Side-on” or “End on” Conformation. *Chem. Biol. Interact.* **2016**, *253*, 100–111.

(7) Dasgupta, N.; Ranjan, S.; Rajendran, B.; Manickam, V.; Ramalingam, C.; Avadhani, G. S.; Kumar, A. Thermal Co-Reduction Approach to Vary Size of Silver Nanoparticle: Its Microbial and Cellular Toxicology. *Environ. Sci. Pollut. Res.* **2016**, *23* (5), 4149–4163.

(8) Helmlinger, J.; Sengstock, C.; Groß-Heitfeld, C.; Mayer, C.; Schildhauer, T. A.; Köller, M.; Epple, M. Silver Nanoparticles with Different Size and Shape: Equal Cytotoxicity, but Different Antibacterial Effects. *RSC Adv.* **2016**, *6* (22), 18490–18501.

(9) Xu, X.; Wang, Y.; Wang, H.; Su, H.; Mao, X.; Jiang, L.; Liu, M.; Sun, D.; Hou, S. Synthesis of Triangular Silver Nanoprisms and Studies on the Interactions with Human Serum Albumin. *J. Mol. Liq.* **2016**, *220*, 14–20.

(10) Maji, A.; Beg, M.; Mandal, A. K.; Das, S.; Jha, P. K.; Hossain, M. Study of the Interaction of Human Serum Albumin with Alstonia Scholaris Leaf Extract-Mediated Silver Nanoparticles Having Bactericidal Property. *Process Biochem.* **2017**, *60*, 59–66.

(11) Zolghadri, S.; Saboury, A. A.; Golestani, A.; Divsalar, A.; Rezaei-Zarchi, S.; Moosavi-Movahedi, A. A. Interaction between Silver Nanoparticle and Bovine Hemoglobin at Different Temperatures. *J. Nanoparticle Res.* **2009**, *11* (7), 1751–1758.

(12) Saeb, A. T. M.; Alshammari, A. S.; Al-Brahim, H.; Al-Rubeaan, K. A. Production of Silver Nanoparticles with Strong and Stable Antimicrobial Activity against Highly Pathogenic and Multidrug Resistant Bacteria. *Sci. World J.* **2014**, *2014*, 1–9.

(13) Bhunia, A. K.; Kamilya, T.; Saha, S. Silver Nanoparticle-Human Hemoglobin Interface: Time Evolution of the Corona Formation and Interaction Phenomenon. *Nano Converg.* **2017**, *4* (1), 28.

(14) Li, X.; Yang, Z.; Peng, Y. The Interaction of Silver Nanoparticles with Papain and

Bromelain. *New J. Chem.* **2018**, *42* (7), 4940–4950.

(15) Wang, G.; Hou, H.; Wang, S.; Yan, C.; Liu, Y. Exploring the Interaction of Silver Nanoparticles with Lysozyme: Binding Behaviors and Kinetics. *Colloids Surfaces B Biointerfaces* **2017**, *157*, 138–145.

(16) Nayak, P. S.; Borah, S. M.; Gogoi, H.; Asthana, S.; Bhatnagar, R.; Jha, A. N.; Jha, S. Lactoferrin Adsorption onto Silver Nanoparticle Interface: Implications of Corona on Protein Conformation, Nanoparticle Cytotoxicity and the Formulation Adjuvanticity. *Chem. Eng. J.* **2019**, *361*, 470–484.
